# γδ T-cell autoresponses to ectopic membrane proteins: a new type of pattern recognition

**DOI:** 10.1038/s41423-025-01258-x

**Published:** 2025-02-13

**Authors:** Hongqin You, Xiangjin Zhang, Hui Chen, Chang Liu, Da Teng, Jiajia Han, Ming Chen, Yongsheng Pang, Jianmin Zhang, Menghua Cai, Yueqi Zhao, Qingqing Dong, Shuli Wang, Yi Xu, Yu Hu, Peng Dong, Wei He

**Affiliations:** 1 Department of Immunology, CAMS Key Laboratory T-Cell and Cancer Immunotherapy, Institute of Basic Medical Sciences, Chinese Academy of Medical Sciences and School of Basic Medicine, Peking Union Medical College, State Key Laboratory of Common Mechanism Research for Major Diseases, Beijing, 100005 China; 2 Beijing Jiadehe Cell Therapy Technology Co., Ltd, Beijing, China; 3 Changzhou Xitaihu Institute for Frontier Technology of Cell Therapy, Changzhou, Jiangsu 213000 China

**Keywords:** γδ T-cell, Ectopic proteins, Pattern recognition, Stress condition, Immunotherapy, Gammadelta T cells, Pattern recognition receptors

## Abstract

T-cell receptor (TCR) γδ-expressing cells are conserved lymphocytes of innate immunity involved in first-line defense and immune surveillance. TCRγδ recognizes protein/nonprotein ligands without the help of the major histocompatibility complex (MHC), especially via direct binding to protein ligands, which is dependent primarily on the δ chain complementary determining region 3 (CDR3δ). However, the mechanism of protein‒antigen recognition by human γδ TCRs remains poorly defined. We hypothesize that γδ TCRs recognize self-proteins expressed ectopically on the cell membrane that are derived from intracellular components under stress. Here, we mapped 16 intercellular self-proteins among 21,000 proteins with a huProteinChip as putative ligands for Vδ1/Vδ2 TCRs, 13 for Vδ1 TCRs and 3 for Vδ2 TCRs. Functional tests confirmed that ectopic nucleolin (NCL) is a ligand for the Vδ1 TCR, whereas protein-glutamine γ-glutamyltransferase K (TGM1) is a ligand for the Vδ2 TCR. In the context of radiation exposure, the ectopic expression of intracellular proteins on the tumor cell surface is related to the increased antitumor cytotoxicity of γδ T cells both in vitro and in vivo. In conclusion, the recognition of intracellular proteins that are ectopically expressed on somatic cells by human γδ TCRs is a basic interaction mechanism that enables new types of immune pattern recognition and a novel γδ TCR-ligand-based strategy for tumor immunotherapy.

## Introduction

In contrast to conventional αβ T cells, human γδ T cells possess several characteristics of innate immune cells [1–7], such as limited diversity and specificity of γδ T-cell receptors (TCRs) for only certain antigens, without major histocompatibility complex (MHC) restriction [8, 9]. In recent decades, research on the mechanism of antigen recognition by γδ TCRs has focused on MHC-related molecules, as well as foreign lipids, glycans and phosphates [4, 10–14]. However, few γδ TCRs specific for protein antigens, which are more diverse and stable in structure than nonpeptidic antigens are well characterized. Previously, we reported CDR3δ-dependent direct binding of the γδ TCR to its protein ligand [1, 15, 16] and identified human mutS homolog 2 (hMSH2) as the ligand for the Vδ2 TCR. hMSH2 is a DNA mismatch repair protein expressed in the nucleolus and ectopically expressed on the cell membrane under stress conditions; this protein is incidentally captured by the CDR3δ peptide designed by our group [17]. We also found that human γδ TCRs primarily use conserved flanking regions of both the CDR3δ2 and CDR3δ1 sequences to bind ligands, which are limited in number and share some of the same motifs in the gene sequence [16, 18]. Since then, we hypothesized that stress-induced ectopic expression of intracellular self-proteins might generate a potential pool of self-antigens for the γδ TCR repertoire. In other words, γδ TCRs recognize intracellular self-proteins on the somatic cell membrane as nonself antigens under stress. In this simple way, γδ TCRs can sense damage to somatic cells and rapidly respond to a series of factors, including cytotoxicity and cytokine production, possibly resulting in the recognition of an innate γδ T-cell-mediated damage-associated molecular pattern (DAMP). Here, we report our findings to confirm this hypothesis.

## Results

### CDR3δ can specifically bind to tumors

Our previous studies demonstrated that the antigen specificity of human γδ TCRs primarily depends on the conserved region in CDR3δ [16, 18]. Two dominant CDR3δ sequences have been identified: a GTM from a Vδ1 TCR expressed by gastric tumor-infiltrating γδ T cells [16] and OT3 from a Vδ2 TCR expressed by epithelial ovarian cancer-infiltrating γδ T cells [15]. Two types of γδ TCR-expressing cells based on these two CDR3δ sequences exhibited strong antitumor activities both in vitro and in vivo [19, 20].

**Fig. 1:**
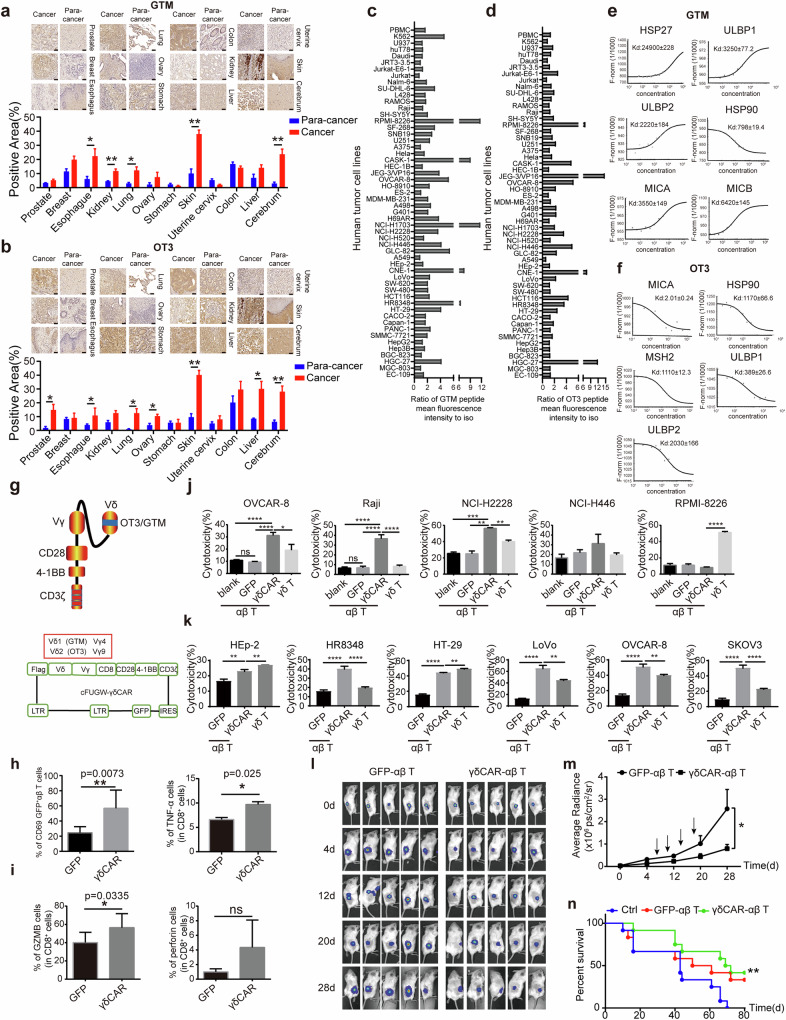
Two CDR3δ peptides, GTM and OT3, with tumor binding specificity served as probes to screen the protein ligands of human γδ TCRs. a and b. Immunohistochemical staining of a tumor tissue array with the biotin-labeled GTM peptide **a** and biotin-labeled OT3 peptide **b** followed by HRP-conjugated streptavidin. The binding was visualized using DAB as the substrate (brown) (400×). Cancer: tumor tissue; paracancerous: paracarcinoma tissues. Scale bar, 50 μm. GTM and OT3 can specifically bind to several tumor cell lines. Fifty-three types of human tumor cell lines were stained with biotinylated (bio)-GTM **c** or (bio)-OT3 peptide **d** and APC-conjugated streptavidin. The binding activity was measured by flow cytometry. The flow data were standardized by the ratio between the mean fluorescence intensity of the peptide conjugate and the secondary antibody alone. The binding strength of the synthetic peptide was compared with that of normal human peripheral blood mononuclear cells (PBMCs). MST analysis of the binding activities of the GTM peptide **e** and OT3 peptide **f** to known γδ TCR-recognized stress-inducible ligands. The Kd value for each protein was calculated. **g** Schematic diagram of OT3- and GTM-γδ CAR molecules. A single-chain Vγ9-Vδ2 or Vγ4-Vδ1 structure, which is composed of a Vδ2 TCR with the tumor-specific CDR3δ sequence OT3 or Vδ1 TCR with the tumor-specific CDR3δ sequence GTM, forms the extracellular region of the γδ CAR. The transmembrane and intracellular segments of a classical third-generation CAR (CD8-CD28-4-1BB-CD3ζ) were linked to the Vγ-Vδ sequence. γδ CARs were cloned and inserted into the lentiviral vector cFUGW. Flow cytometry was used to assess the expression of the activation marker CD69, the secretion of TNF-α **h** and the cytotoxicity-related molecules granzyme B and perforin **i** in γδ CAR-T cells after coculture with OVCAR-8 cells in vitro. The statistical data are representative of three independent experiments and are expressed as the means ± SDs. *, *p* < 0.05; **, *p* < 0. 01. Cytotoxicity of OT3 CAR-T cells **j** and GTM CAR-T cells **k** in different tumor cells in vitro. Cytotoxicity was analyzed with an LDH cytotoxicity detection kit. The data are representative of three independent experiments and are expressed as the means ± SDs. OT3 CAR-αβ T cells exhibit strong cytotoxic activity against tumors in an NSG mouse model. OVCAR-8-transplanted NSG mice were treated with GFP-αβ T cells or OT3 CAR-αβ T cells. Tumor growth in every mouse at each time point was measured by assessing the radiance of the tumor cells **l**. The tumor growth curve **m** and survival curve **n** of each group are shown

In the present study, we synthesized two biotin-labeled CDR3δ peptides, GTM and OT3, and performed immunohistochemistry (IHC) with MC246a tumor tissue chips containing 12 different organ tumor tissues and matched paracarcinoma tissues. The results showed that both the GTM (Fig. 1a) and OT3 (Fig. 1b) peptides could bind to various different tumor tissues compared with the paracarcinoma controls. Flow cytometric analysis of 50 human tumor cell lines (Supplementary Data Table 2) revealed that although the binding patterns were quite different, both peptides bound to various tumor cell lines but showed low binding activities to human PBMCs, which served as a normal control (Fig. 1c, d). Human γδ T cells can directly recognize stress-induced proteins, such as the UL16 binding protein (ULBP) family [21], MHC I-related A/B (MICA/B) [22], MSH2 [17], and heat shock protein (HSP) family proteins [23], through both the γδ TCR and the natural killer (NK) cell receptor NKG2D. An MST revealed that the GTM and OT3 peptides could also bind to some of these known ligands (Fig. 1e, f).

Next, we designed two CAR molecules with single-chain Vγ9-Vδ2 (OT3) and Vγ4-Vδ1 (GTM) as their extracellular domains, which replaced the conventional single-chain Fv fragment (Fig. 1g), to evaluate whether the structure can specifically bind to tumor cells and mediate CAR-T-cell cytotoxicity. The γδ CAR molecule was inserted into the lentiviral vector cFUGW, which contains the CD28 transmembrane domain, and 4-1BB and CD3ζ, which are classic CAR molecules. We packaged the viruses and infected αβ T cells. The expression of exogenous γδ CAR molecules was validated by Western blotting and flow cytometry of the GFP signal (Supplementary Data Fig. 1). When coincubated with tumor cells, γδ CAR-T cells presented increased expression levels of CD69, TNF-α (Fig. 1h) and granzyme B (Fig. 1i). LDH release assays were used to measure the cytotoxicity of γδ CAR-T cells against different tumor cell lines in vitro. OT3 γδ CAR-T cells exhibited stronger cytotoxicity against OVCAR-8, Raji and NCI-H2228 cells (Fig. 1j), and GTM γδ CAR-T cells exhibited stronger cytotoxicity against HR8348, LoVo, OVCAR-8 and SKOV3 cells (Fig. 1k).

To further confirm the specific targeting of γδ CAR to tumor-related proteins, NOD-Prkdc^em26cas9d52^Il2rg^em26cas9d22^Nju (NCG) mice transplanted with OVCAR-8 cells were used as a model. Compared with control T cells, OT3 γδ CAR-T cells exhibited stronger cytotoxic activity against tumor cells in vivo, as tumor growth was significantly inhibited (Fig. 1l, m), and the survival time of the tumor-bearing mice was significantly prolonged (Fig. 1n). Taken together, our results suggest the specific binding and functions of CDR3δ1 GTM and CDR3δ2 OT3 to tumor-related antigens, which can serve as probes to screen the protein ligands of human γδ TCRs.

### Mapping protein ligands for γδ TCRs

**Fig. 2:**
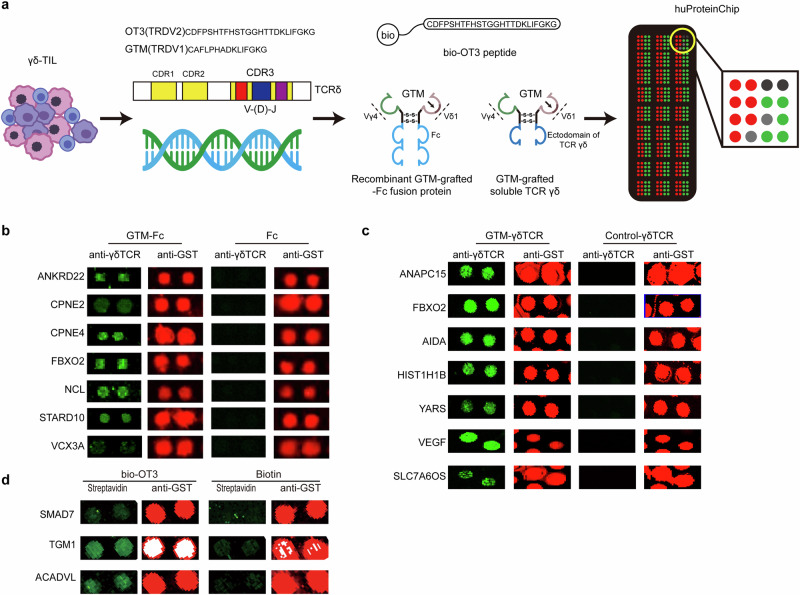
Screening the potential protein ligands of γδ T cells via a tumor-specific CDR3δ sequence strategy. **a** Schematic diagram of the tumor-specific CDR3δ sequence strategy. Tumor-infiltrating γδ T cells were cultured, and their CDR3δ sequences were analyzed. Two biotin-CDR3δ peptides, GTM and OT3, were synthesized according to the dominant sequences, and their tumor binding specificities were verified. A recombinant GTM-grafted Vγ4δ1-Fc fusion protein/GTM-grafted soluble γδ TCR and biotin-OT3 peptide were subsequently used as probes to screen novel ligands of γδ T cells via the huProteinChip. b-d. Screening the potential protein ligands of γδ T cells via recombinant GTM-grafted Vγ4δ1-Fc fusion protein **b** GTM-grafted soluble γδ TCR **c** and biotin-labeled OT3 peptide **d** with the huProteinChip. The green fluorescence represents the specific binding signals of the probes, and the red fluorescence represents the protein loading control detected by anti-GST

On the basis of two tumor-specific CDR3δ sequences derived from γδTILs, we then applied a recombinant GTM-grafted Vγ4δ1-Fc fusion protein and GTM-grafted soluble γδ TCR as probes to identify the potential protein ligands for the Vδ1 TCR, and we used a biotin-OT3 peptide to identify the potential protein ligands of the Vδ2 TCR; these ligands were identified through high-throughput screening via a HuProt™ human proteome microarray, which provides the largest number of unique human proteins known to be included on a single slide, allowing thousands of interactions to be profiled in a high-throughput manner [24] (Fig. 2a). Among the 21,000 proteins, 13 proteins were found to specifically bind to the GTM-grafted Vγ4δ1-Fc fusion protein and GTM-grafted soluble γδ TCR (Fig. 2b, c), whereas 3 proteins were found to bind to the biotin-labeled OT3 peptide (Fig. 2d, Supplementary Data Table 3). These results suggest that γδ TCRs recognize a limited variety of protein ligands in a pattern recognition-like manner. Impressively, all of these identified potential protein ligands are physiologically located in an intercellular compartment—either the nucleus or the cytosol.

### Ectopic NCL, a ligand for the Vδ1 TCR

Next, further studies were performed to functionally verify the protein ligands of γδ T cells. We validated hMSH2, a protein that exhibits stress-induced ectopic expression, as a new functional protein ligand for the Vδ2 TCR. hMSH2 is recognized by γδ T cells to trigger cytotoxicity against tumor cells [17]. Thus, we selected NCL, a candidate ligand for the Vδ1 TCR, to validate whether this new recognition mechanism is suitable for all the major human γδ T-cell subsets, both Vδ1 and Vδ2 T cells.

NCL, a highly conserved RNA-binding protein with multiple functions in several cellular processes, such as chromatin remodeling, rRNA synthesis, mRNA processing, ribosome assembly, and nucleocytoplasmic transport [25], is expressed mainly in the nucleus. However, under conditions of cellular stress or in tumor cells, NCL is upregulated and translocated to the cytosol and cell surface membrane to display danger signals and induce antitumor responses [26].

**Fig. 3:**
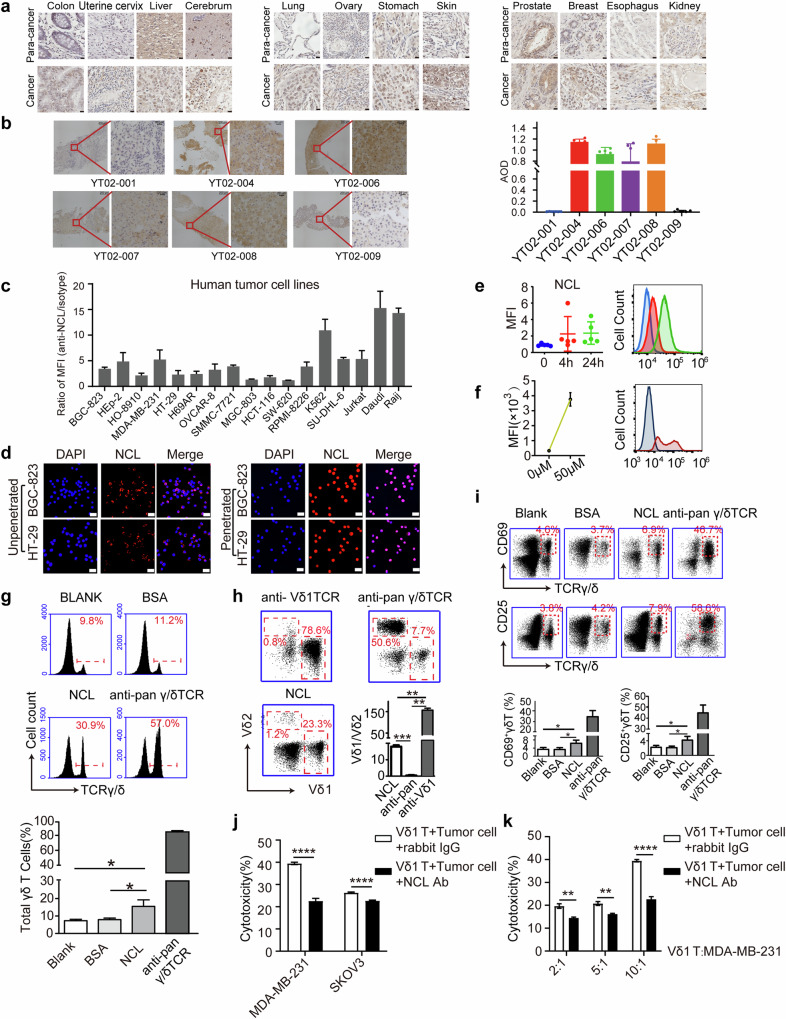
Ectopically expressed NCL serves as a stress-induced ligand of Vδ1 T cells. **a** Immunohistochemical staining of a human tumor tissue microarray with anti-NCL antibodies. Cancer: tumor tissues; paracarcinoma: paracarcinoma tissues. Scale bar, 20 μm. **b** Immunohistochemical staining of NCL in tumor biopsy samples from six HCC patients. The average optical density (AOD) value was measured to represent the expression level of NCL. Six fields of view were selected for each sample. The statistical data for each sample are shown on the right. Scale bar, 20 μm. **c** Flow cytometric analysis of ectopic NCL protein expression on the surface of different human tumor cell lines. PBMCs served as the control. **d** Immunofluorescence assay examining the expression of NCL proteins on the cell surface and in the intracellular compartments of gastric adenocarcinoma BGC-823 cells and colorectal cancer HT-29 cells. Scale bar, 20 μm. **e** Ectopic expression of NCL in menadione-treated 293 T cells was upregulated in a time-dependent manner. Three independent experiments were performed for statistical analysis (left), and representative results (right) are shown. **f** Short-term high-dose menadione treatment also upregulated the ectopic expression of NCL in 293 T cells. Three independent experiments were conducted for statistical analysis (left), and representative results are shown (right). Flow cytometric analysis of the proliferation **g**, subtype **h** and activation marker expression **i** of γδ T cells isolated from PBMCs and stimulated with the recombinant NCL protein. Three independent experiments were performed for statistical analysis, and representative results are shown. The anti-NCL antibody partially blocked the cytotoxic effects of Vδ1 T cells on different tumor cells **j** and different effect‒target ratios **k**. Cytotoxicity was examined via an LDH assay, and Vδ1 T cells expanded with the anti-Vδ1 TCR were used as effectors. For all the flow cytometric data, representative plots are shown. The error bars show the mean values with SEMs for at least three independent experiments. *P* values were calculated via two-tailed unpaired *t* tests with Welch’s correction; *, *p* < 0.05; **, *p* < 0.01; ***, *p* < 0.001

Although the direct binding of GTM, GTM-Fc or γ4δ1(GTM)-TCR to the recombinant NCL protein was not detected by the MST assay, which may be due to the lack of necessary posttranslational modifications or chaperones of the recombinant NCL protein in vitro, the interaction between GTM and the NCL-Fc fusion protein was confirmed by an immunoprecipitation assay (Supplementary Data Fig. 2). Immunohistochemistry assays with MC246a tumor tissue chips were conducted to examine the levels of NCL expression in tumor tissues, and the results revealed that NCL expression was upregulated in a variety of human tumor tissues compared with their paracarcinoma control tissues (Fig. 3a). The immunohistochemical results also revealed high expression of NCL in tumor biopsy samples from 4 of the 6 patients with primary hepatocellular carcinoma (Fig. 3b, Supplementary Data Fig. 3). Flow cytometry was also performed to detect the ectopic expression of NCL on the surface of 17 different human tumor cell lines. Despite the variations among the tumor cell lines, some exhibited high levels of ectopic NCL expression on the cell surface (Fig. 3c). We also performed immunofluorescence assays to examine both the ectopic expression and the nuclear expression of NCL in BGC-823 cells and HT-29 cells (Fig. 3d). The results revealed that most of the NCL proteins were in the nucleus, but some were ectopically expressed on the cell surface.

In addition to tumors, to determine whether the ectopic expression of NCL results from cell stress, we established a DNA damage-induced cell stress model with menadione [8] (Supplementary Data Fig. 4a–4c), which is widely used as an oxidative injury-inducing agent that causes reduced cell viability, increased poly ADP‒ribose (PAR) accumulation, and increased sensitivity to cytotoxic effects [7, 22]. Menadione treatment caused a significant increase in the expression of stress-inducible γδ TCR ligands, including ULBP1, ULBP4, hMSH2 and HSP70 (Supplementary Data Fig. 4d), as well as NCL (Fig. 3e), in 293 T cells. In tumor cell lines, menadione treatment-induced stress also significantly upregulated the expression of NCL (Fig. 3f) and hMSH2 (Supplementary Data Fig. 4e) on the cell surface. Collectively, these findings suggest that NCL is a stress-inducible molecule that is ectopically expressed in a variety of tumor cells or normal cells under stress conditions.

To further verify whether NCL is a ligand of γδ T cells, we purified NCL proteins to amplify human γδ T cells from PBMCs in vitro. The results showed that the recombinant NCL protein could specifically stimulate the proliferation of γδ T cells, resulting in a significant increase in the γδ T-cell percentage (Fig. 3g). In particular, we found that NCL predominantly stimulated the proliferation of Vδ1 T cells rather than Vδ2 T cells (Fig. 3h). The expression of the activation markers CD69 and CD25 on γδ T cells was also significantly increased (Fig. 3i). Repeated experiments using sorted γδ T cells (Supplementary Data Fig. 5) but not PBMCs yielded similar results (Supplementary Data Fig. 6). An in vitro Vδ1 T-cell expansion method was established for Vδ1 T cells with purities above 80% (Supplementary Data Fig. 7). Although there was no significant increase in the secretion of IFN-γ or IL-2 by Vδ1 T cells after NCL stimulation (Supplementary Data Fig. 8), blocking NCL with anti-NCL antibodies significantly decreased the cytotoxicity of Vδ1 T cells to MDA-MB-231 and SKOV3 cells (Fig. 3j, k).

Taken together, these findings confirmed that NCL is ectopically expressed on tumor cells or normal cells under conditions of stress and that NCL is a stress-inducible protein ligand of Vδ1 T cells. In addition to NCL, FBXO2, which is highly expressed in various human colorectal cancer cell lines, also specifically binds to the GTM-grafted Vγ4δ1-Fc fusion protein in BLI (Supplementary Data Fig. 9), suggesting a potential ectopic protein ligand for the Vδ1 TCR for which further validation is needed.

### Antitumor strategies targeting ectopic stress-inducing ligands

**Fig. 4:**
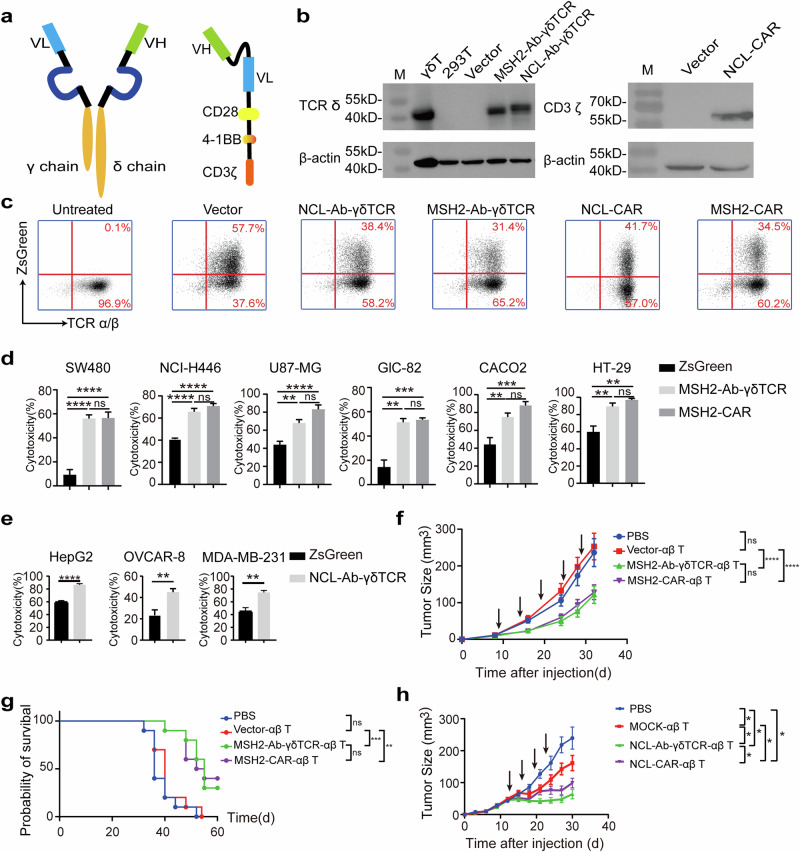
Construction and functional verification of Ab-γδ TCR/CAR-T cells that target ectopically expressed hMSH2 and NCL. **a** Schematic of Ab-γδ TCR (left) and CAR (right) molecules. **b** Western blotting was performed to evaluate the expression of Ab-γδ TCRs and CARs in 293 T cells with anti-TCR δ chain and anti-CD3ζ antibodies. **c** Representative flow cytometric plots of the ZsGreen signal showing the transduction efficacy. RTCA was performed to measure the cytotoxicity of hMSH2 Ab-γδ TCR/CAR-T cells **d** and NCL-Ab-γδ TCR-T cells **e** against tumor cells in vitro. The plot shows the statistical data at 24 h. The data are representative of three independent experiments and are expressed as the means ± SDs. *, *p* < 0.05; **, *p* < 0.01; ***, *p* < 0.001; ****, *p* < 0.0001; ns, *p* > 0.05, not significant. hMSH2-Ab-γδ TCR-T cells and hMSH2-CAR-T cells exhibited strong cytotoxic activity against tumor cells in the B-NDG mouse model. B-NDG mice implanted with SW480 cells were treated with Vector-αβ T cells, hMSH2-Ab-γδ TCR-T cells, or hMSH2-CAR-T cells. Tumor growth in every mouse at each time point was measured with a Vernier caliper. The tumor growth curve **f** and survival curve **g** of each group are shown. The arrow shows the time point for treatment. **, *p* < 0.01; ***, *p* < 0.001; ****, *p* < 0.0001; ns, *p* > 0.05, not significant. **h** NCL-Ab-γδ TCR-T cells exhibited strong cytotoxic activity against tumor cells in the B-NDG mouse model. B-NDG mice implanted with HepG2 cells were treated with MOCK-T cells, NCL-Ab-γδ TCR-T cells or NCL-CAR-T cells. Tumor growth in every mouse at each time point was measured with a Vernier caliper. The arrow shows the time point for treatment

For the ectopic expression of NCL on the tumor cell membrane, we designed two types of genetic T cells on the basis of specific antibodies targeting NCL to validate their antitumor efficacy: classic CAR-T cells, which had the CD28, 4-1BB and CD3 domains, and Ab-γδ TCR-T cells, in which the VL and VH fragments of the anti-NCL or anti-hMSH2 antibodies were used to replace the V fragment of the γ and δ chains, respectively, in the γδ TCR (Fig. 4a). CAR-T cells and Ab-γδ TCR-T cells targeting hMSH2 were used as positive controls. The expression of the Ab-γδ TCR molecule and CAR molecule in 293 T cells was validated by Western blotting (Fig. 4b). After viral transduction, exogenous CAR^+^ and Ab-γδ TCR^+^ αβ T cells were analyzed by evaluating the ZsGreen signal via fluorescence microscopy (Supplementary Data Fig. 10) and flow cytometry (Fig. 4c). Both CAR-T cells and Ab-γδ TCR-T cells exhibited proliferative activity in vitro comparable to that of unmodified αβ-T cells (Supplementary Data Fig. 11). We measured the cytotoxicity of CAR-T cells and Ab-γδ TCR-T cells against different tumor cell lines via RTCA. hMSH2-Ab-γδ TCR-T cells exhibited stronger cytotoxicity against various tumor cell lines with much higher levels of ectopic surface hMSH2 expression, including SW480, NCI-H446, U87-MG, GLC-82, Caco2 and HT-29 cells (Fig. 4d, Supplementary Data Fig. 12a, Supplementary Data Fig. 13a). NCL-Ab-γδ TCR-T cells also exhibited stronger cytotoxicity against HepG2, OVCAR-8, and MDA-MB-231 cells (Fig. 4e, Supplementary Data Fig. 12b), which presented much higher ectopic NCL expression (Supplementary Data Fig. 13b). Furthermore, hMSH2-Ab-γδ TCR-T cells exhibited significantly greater cytotoxicity against NCI-H520 cells overexpressing hMSH2 (Supplementary Data Fig. 14a, b). Similar results were observed via LDH cytotoxicity assays for the cytotoxicity of NCL-Ab-γδ TCR-T cells against NCL-overexpressing A549 cells (Supplementary Data Fig. 14c, d).

To further confirm the antitumor efficacy of hMSH2/NCL-targeted CAR-T cells and Ab-γδ TCR-T cells in vivo, we established tumor models in NOD. CB17-PrkdcscidIl2rgtm1/Bcgen (B-NDG) mice generated via the subcutaneous injection of SW480 cells or HepG2 cells. hMSH2-targeted CAR-T cells and Ab-γδ TCR-T cells significantly inhibited the growth of tumors (Fig. 4f) and prolonged the survival of tumor-bearing mice (Fig. 4g). Similarly, NCL-CAR-T cells and NCL-Ab-γδ TCR-T cells significantly inhibited tumor growth in a HepG2 tumor-bearing mouse model, while NCL-CAR-T cells showed slightly better efficacy than NCL-Ab-γδ TCR-T cells in vivo (Fig. 4h). These results suggest that, similar to hMSH2, NCL can be ectopically expressed on the surface of tumor cells and could serve as a target for tumor immunotherapy.

### Ectopic TGM1, a ligand for the Vδ2 TCR

Similar to our verification of NCL as an ectopically expressed ligand of Vδ1 TCRs, we also verified that TGM1 is a novel ectopically expressed ligand of the Vδ2 TCR.

**Fig. 5:**
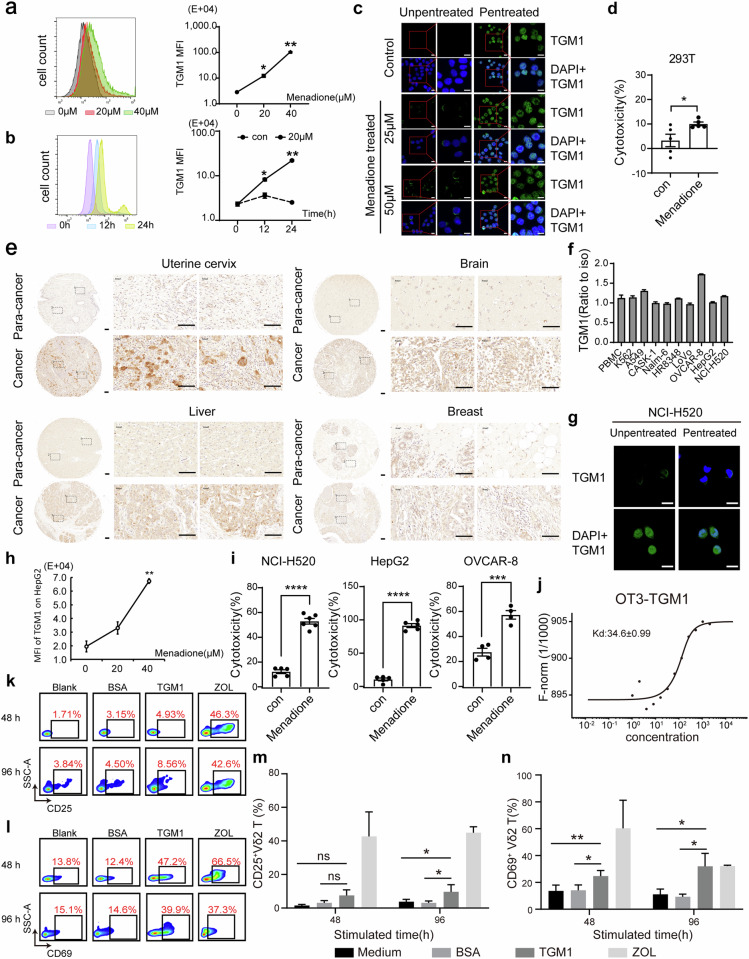
Ectopically expressed TGM1 served as a stress-induced ligand of Vδ2 T cells. Ectopic expression of TGM1 in menadione-treated 293 T cells was upregulated in a dose-dependent **a** and time-dependent **b** manner. **c** Unpenetrated 293 T cells presented significantly upregulated cell membrane expression of TGM1 after menadione treatment according to confocal microscopy. Scale bar, 10 μm. **d** Upregulated ectopic TGM1 expression increased the cytotoxicity of γδ T cells to menadione-treated 293 T cells. **e** TGM1 expression in various human tumor tissues was upregulated compared with that in paracarcinoma control tissues. Scale bar, 100 μm. Flow cytometry **f** and confocal microscopy **g** results indicating that TGM1 was ectopically expressed on the surface of OVCAR-8 and NCI-H520 cells. Scale bar, 10 μm. Menadione triggered a significant increase in the ectopic expression of TGM1 on the surface of HepG2 cells **h** in a dose-dependent manner. Menadione treatment also significantly increased the sensitivity of NCI-H520, HepG2 and OVCAR-8 cells to γδ T-cell cytotoxicity **i**. **j** MST analysis of the binding activity of the OT3 peptide to TGM1. **k, l, m, n** Flow cytometric analysis of the activation of Vδ2^+^ T cells stimulated with the recombinant TGM1 protein for 48 h and 96 h. CD25 **k**. CD69 **l**. *, *p* < 0.05; **, *p* < 0.01; ***, *p* < 0.001; ****, *p* < 0.0001; ns, *p* > 0.05, not significant

TGM1 is a transglutaminase 1 enzyme that catalyzes the addition of an alkyl group from alkylamine residues to glutamine residues, forming alkylglutamine residues in proteins [27]. We found that menadione triggered ectopic TGM1 expression in normal 293 T cells in a dose (Fig. 5a)- and time (Fig. 5b)-dependent manner. Unpenetrated 293 T cells presented significantly upregulated cell membrane expression of TGM1 after menadione treatment, as shown by confocal microscopy (Fig. 5c), and ectopic expression of TGM1 significantly increased the cytotoxicity of Vδ2 γδ T cells to 293 T cells (Fig. 5d).

We also examined TGM1 expression in tumor tissues and tumor cell lines and found that ectopic expression of TGM1 was upregulated in some tumor tissues (Fig. 5e) and some tumor cell lines (Fig. 5f, g). Consistent with the results in menadione-treated 293 T cells, menadione treatment resulted in a significant increase in the ectopic expression of TGM1 on the surface of tumor cells and significantly increased the cytotoxicity of γδ T cells to tumor cell lines (Fig. 5h, i).

To further verify whether TGM1 is a ligand of γδ T cells, we demonstrated the specificity of the binding of OT3 and TGM1 via an MST assay (Fig. 5j). Moreover, we amplified human γδ T cells isolated from PBMCs in vitro. The results showed that the recombinant TGM1 protein could specifically upregulate the expression of the activation markers CD69 and CD25 on Vδ2^+^ γδ T cells (Fig. 5k–n). Taken together, these findings confirmed that TGM1 could be ectopically expressed on the surface of tumor cells and cells after menadione treatment and serve as a ligand for Vδ2 γδ T-cell activation and cytotoxicity.

### Ligand-induced γδ T-cell cytotoxicity

Although γδ T cells are believed to recognize a wide range of structurally different ligands, very few true protein ligands have been identified as being recognized by γδ TCRs. Functionally, without negative selection during thymus development, γδ T cells exploit their limited diversity to recognize stress-induced self-protein antigens, including MHC class I chain-related gene A/B (MICA/B) [7, 22], UL-16-binding proteins (ULBPs) [21] and heat shock proteins (HSPs) [23], as well as hMSH2. The finding that γδ T cells may recognize a series of stress-induced ectopic protein ligands prompted us to explore the importance of these ectopic proteins in the γδ T-cell-mediated antitumor immune response. We conducted a systematic analysis of the effects of the known stress-induced ligands described above on different tumor cell lines and γδ T-cell cytotoxicity in vitro.

**Fig. 6:**
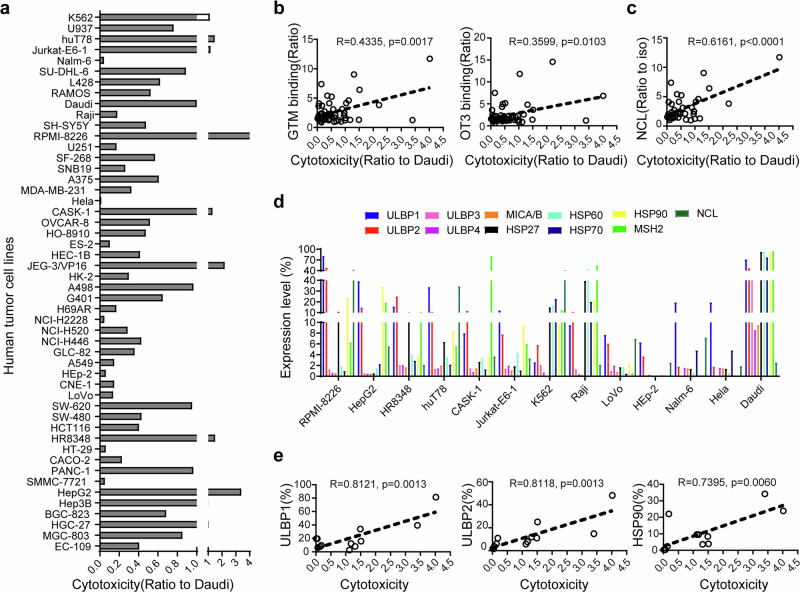
The cytotoxicity of human γδ T cells against tumor cell lines was positively correlated with the ectopic expression of γδ T-cell ligands on tumor cells. A systematic cytotoxicity assay of human γδ T-cell responses to 50 human tumor cell lines was performed via an LDH assay (E:T = 10:1). **a** The cytotoxicity of γδ T cells against different target tumor cells is shown as ratios and normalized to their cytotoxicity against Daudi cells. Correlation analysis was performed with the 50 human tumor cell lines to analyze the correlation between their binding activities to GTM/OT3 **b** or their ectopic expression of NCL **c** with their sensitivity to γδ T-cell cytotoxicity. Flow cytometry was used to detect the expression of 11 stress-inducible ligands that are recognized by γδ T cells in 13 different human tumor cell lines, and the percentage of positive cells (compared with that of the iso cells) is displayed to indicate the expression level of certain stress-inducible ligands **d**. The expression of 3 of these ligands, ULBP1, ULBP2 and HSP90, was positively correlated with γδ T-cell cytotoxicity **e**

The results of the cytotoxicity assay revealed that γδ T cells exhibited significant cytotoxicity against 71.2% of 50 human cell lines without tumor type specificity (Fig. 6a, Supplementary Data Table 4). Correlation analysis revealed a significant positive correlation between the cytotoxicity of γδ T cells and the binding activity of tumor cells to GTM and OT3 peptides (Fig. 6b), as well as with the ectopic expression of NCL (Fig. 6c).

Flow cytometry was used to assess the expression of all 11 stress-inducible ligands that are recognized by γδ T cells on 13 different human tumor cell lines (Fig. 6d), and correlation analysis of the expression of these ligands and γδ T-cell cytotoxicity was performed. The results revealed that the expression of ULBP1, ULBP2 and HSP90 was positively correlated with γδ T-cell cytotoxicity (Fig. 6e), whereas that of the other genes was not (Supplementary Data Fig. 15). Furthermore, we analyzed the expression levels of stress-induced ligands on the surface of four pairs of tumor cell lines derived from the same tissue type; these cell lines included HeLa and CASK-1 cells (cervical carcinoma), Raji and Daudi cells (B lymphoma), LoVo and HR8348 cells (colorectal carcinoma), and SMMC-7721 and HepG2 cells (hepatocellular carcinoma), and these cell types were significantly different in their sensitivities to γδ T-cell cytotoxicity. Compared with those in the γδ T-cell-sensitive cell lines, the expression levels of most stress-inducible proteins in the γδ T-cell-insensitive cell lines were significantly lower (Supplementary Data Fig. 16), confirming that the stress-induced ectopic expression of proteins was closely associated with the cytotoxicity of human γδ T cells to tumor cells.

### Unregulated ectopic proteins under radiation treatment

**Fig. 7:**
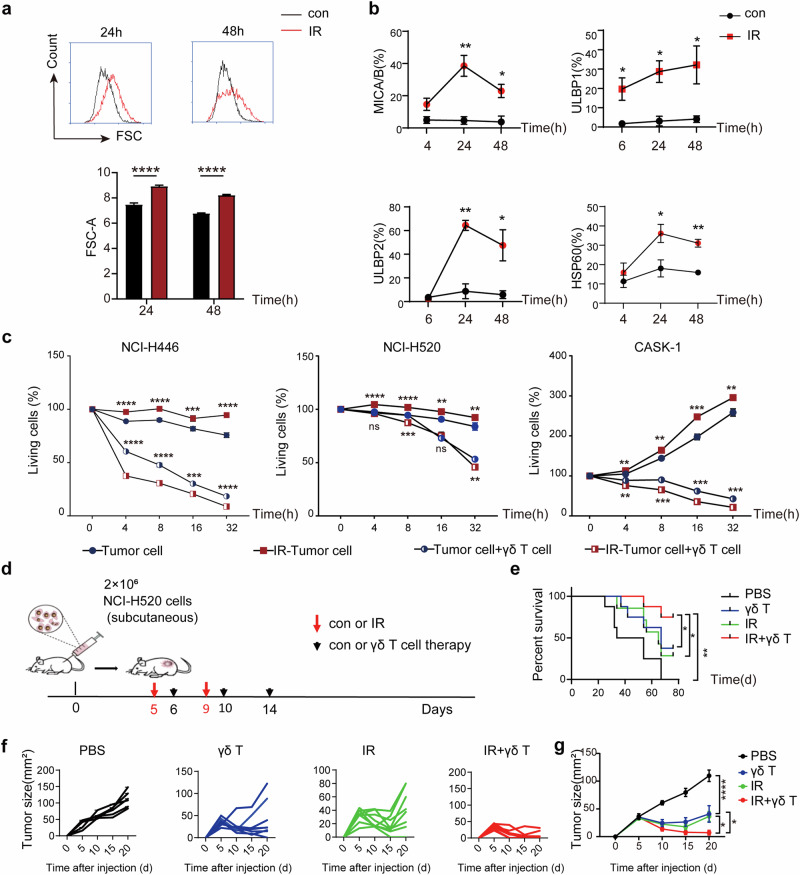
The combination of γδ T-cell therapy with radiation increased the antitumor efficacy of human γδ T-cell immunotherapy by increasing the ectopic expression of stress-induced ligands on irradiated tumor cells. **a** Irradiation exposure can lead to morphological changes in tumor cells. After exposure to 5 Gy radiation, the FSC of NCI-H520 cells significantly increased from 24 h to 48 h, indicating that the cells were in a state of stress. **b** Irradiation upregulated the ectopic expression of the ligands ULBP1, ULBP2, HSP60 and MICA/B on NCI-H520 cells in vitro. Flow cytometric results showing ligand expression on the tumor cell membrane before and at different time points after irradiation. **c** The cytotoxicity of human γδ T cells to irradiated tumor cells was significantly increased. The cytotoxicity of γδ T cells to three kinds of tumor cells treated with or without 5 Gy ionizing radiation was recorded via RTCA. **d** Design of the in vivo experiment in mouse transplanted tumor models. γδ T-cell adoptive immunotherapy alone or in combination with radiotherapy was compared in this study. The combination of immunotherapy with low-dose radiation therapy prolonged survival **e** and promoted tumor regression in tumor-bearing mice. The tumor growth of each mouse in each group is shown **f**, and the average value of all the groups is shown **g** *, *p* < 0.05; **, *p* < 0.01; ***, p < 0.001 (ANOVA). For survival curves, ANOVA and the log-rank (Mantel‒Cox) test were performed. *, *p* < 0.05; **, *p* < 0.01; ***, p < 0.001

Radiotherapy is one of the major cancer treatment strategies and induces cellular stress through DNA damage, subcellular organelle damage, and reactive oxygen species generation [28]. We applied a radiation cell model to simulate radiotherapy-induced cell stress to observe the changes in ectopic protein expression and subsequently the changes in γδ T-cell cytotoxicity against target cells. The results revealed that after irradiation, tumor cells exhibited obvious morphological changes (Fig. 7a), and the expression levels of the stress-inducible proteins ULBP1, ULBP2, HSP60 and MICA/B were also significantly increased in irradiated tumor cells (Fig. 7b, Supplementary Data Fig. 17); these effects were accompanied by the significantly increased cytotoxicity of human γδ T cells against these irradiated cells in vitro (Fig. 7c, Supplementary Data Fig. 18).

Next, we investigated whether low-dose radiation can increase the antitumor effect of γδ T cells in a transplanted tumor mouse model (Fig. 7d). We found that two ionizing radiation treatments (2 Gy each) at local tumor sites 24 h before the first two γδ T-cell injections significantly increased the sensitivity of tumors to γδ T-cell-mediated antitumor activity. Compared with γδ T cells or radiation treatment alone, combined therapy with γδ T cells and radiation significantly prolonged the survival of tumor-bearing mice and inhibited the growth of tumors (Fig. 7e–g). Overall, our results demonstrate that radiation increases the ectopic expression levels of stress-induced protein ligands on the surface of tumor cells and subsequently promotes the antitumor efficacy of γδ T cells.

## Discussion

In the mid-1980s, γδ T cells were discovered in both mice and humans [29]. During intrathymic development, γδ T cells emerge before αβ T cells at the early stages, and in vivo, most mature γδ T cells home toward mucosal and epithelial tissues, in which γδ T cells play unique roles in first-line responses to infection and tumor immune surveillance via direct cytotoxicity and the secretion of cytokines, such as IFN-γ and IL-17 [1, 8, 30]. Unlike mouse γδ T cells, human γδ T cells are classified into two major subsets according to their δ chain usage. Vδ1 T cells pair with Vδ1 chains to form different Vγ chains, which are preferentially enriched in epithelial tissues, whereas Vγ9Vδ2 T cells, which are not present in mice, are distributed in peripheral lymphoid tissue and account for only 1–5% of circulating T lymphocytes [1, 2, 31]. Vδ1 and Vδ2 T cells are almost completely composed of human γδ T cells, except for a very small number of Vδ3 subsets in the liver.

Without CD4 or CD8 molecules, γδ T cells do not seem to undergo both positive and negative selection during thymus development. The principal antigenic specificity of an immunoglobulin derives from its most diverse CDR3 region of the heavy chain. Owing to the similarity of γδ TCRs and immunoglobulins in direct ligand binding [1], CDR3δ serves as the key determinant for the specificity of γδ TCR antigen binding, with minimal assistance by the γ chain. The critical role of CDR3δ in antigen recognition by γδ T cells has been well confirmed [15]. Owing to the limited diversity of the γδ TCR repertoire [32], γδ TCRs do not generally bind to protein antigens such as peptides presented by MHC molecules in a one-to-one manner [33, 34] but tend to recognize some nonpeptidic antigens, self-MHC-like molecules and stress-induced self-proteins directly. However, the specific recognition pattern of γδ T cells for these protein antigens remains unclear.

One of the core roles of immune recognition is to distinguish between “self” and “nonself”. Innate immune cells, such as epithelial or myeloid cells, respond to foreign and internal environments via the interaction of pattern recognition receptors (PRRs) with PAMPs or DAMPs, and NK cells respond to the abnormal absence of self-MHC I or increased stress-related self-ligands on somatic cells. B cells bind to antigens directly through diverse B-cell receptors (BCRs), and αβ TCRs recognize antigenic peptides and MHC complexes on antigen-presenting cells, both of which are antigen specific. Through structural approaches to examine the roles of γδ TCRs in antigen recognition, we found that γδ TCRs primarily use conserved flanking regions of the CDR3δ sequence to bind ligands [18], suggesting a limited number of γδ TCR ligands.

On the basis of tumor-specific CDR3δ OT3, we previously validated ectopically expressed hMSH2 as a protein ligand of the Vδ2 TCR [21] and hypothesized that stress-induced membrane-expressing proteins serve as “danger signals” to trigger γδ T-cell-mediated responses. In this study, we used two types of probes, a GTM-grafted Vγ4δ1-Fc fusion protein and a biotin-labeled Vδ2 CDR3 OT3 peptide, which basically cover the two main kinds of human TCR cell populations, to screen for additional protein ligands of γδ TCRs. Among the 21,000 proteins, we identified 16 intracellular candidates and validated ectopically expressed NCL and TGM-1 as protein ligands of the Vδ1 and Vδ2 TCRs, respectively. Furthermore, we found that these stress-inducible ligands could be upregulated on the surface of tumor cells after radiation exposure and could then be recognized by γδ T cells or several ligand-targeting engineered CAR-T cells and TCR-T cells, subsequently mediating the “recognition as killing” process to eliminate tumor cells.

A previous report demonstrated that only phosphorylated NCL can interact with the peptide-binding domain of heat shock cognate 70 (HSC70), which is a chaperone, resulting in the ectopic expression of NCL from the intracellular region to the cell surface [35]. NCL undergoes multiple posttranslational modifications, such as phosphorylation, glycosylation, and acetylation, in vivo [25, 36]. The interaction between the recombinant NCL protein and GTM is weak in MST, but immunoprecipitation assays and functional experiments have shown that NCL can stimulate the expansion, activation, and cytotoxic activity of Vδ1 T cells, which indicates that the need for molecular chaperones and posttranslational modifications is highly important for the interaction of cell surface-expressed NCL with Vδ1 T cells.

Answering the fundamental immunological question of how γδ T cells recognize protein antigens is highly important. That is, both the Vδ1 and Vδ2 subsets of human γδ T cells can recognize certain ectopically expressed self-proteins on the cell surface, including NCL and hMSH2, which are produced because of exposure to stress, such as infection or malignant transformation, and play roles as first-line defense mediators and sensors for immunosurveillance.

First, unlike NK cell activation, which involves mutual regulation of activating receptors and inhibitory receptors in a concise and rapid manner via long-term evolution, according to the tissue distribution of γδ T cells, stress-induced ectopic expression of single or multiple proteins leads to a rapid local response of γδ T cells, direct cytotoxicity, or the secretion of cytokines for inflammation and the regulation of subsequent adaptive immune responses.

In contrast to the αβ TCR, the conserved flanking region of the CDR3δ sequence plays a crucial role in antigen recognition, which determines the innate property in which the protein ligands recognized by the γδ TCR are limited. The unique antigen recognition pattern of γδ TCRs to certain ectopically expressed self-proteins can also be considered as a special version of pattern recognition and a supplement to the pattern recognition mechanism.

Previous studies on the recognition of nonpeptide antigens by γδ T cells are limited; however, proteins are the most advanced form of living substances and exhibit greater diversity in their changes and more universal importance in their roles. The recognition of stress-induced protein expression by γδ T cells occurs under a range of different circumstances, including tumors, cellular aging, noninfectious inflammation, infection, intracellular symbiosis, autoimmune diseases, and immune homeostasis imbalance, indicating broader application prospects for γδ T-cell-based immunotherapy.

Taken together, our results indicate that γδ T cells, which have limited TCR diversity, can perform innate-like functions and recognize ectopically expressed intracellular self-proteins on many different cell types as DAMPs under stress conditions. This finding highlights a pioneering antigen recognition mechanism of both Vδ1 and Vδ2 TCRs to protein antigens. Moreover, immunotherapeutic strategies, for example, combination with radiotherapy, which synergistically promotes the potency of γδ T-cell therapy by increasing the expression of stress-induced ectopic proteins, or Ab-TCR-T-cell or CAR-T-cell therapy, which directly targets stress-induced ectopic proteins, can provide new ideas for the treatment of tumors, as well as infection, aging and autoimmune diseases.

## Materials and methods

### Clinical tumor samples

Liver puncture biopsy samples were obtained from 6 hepatocellular carcinoma patients who participated in an investigator-initiated trial (NCT04032392). The trial was approved by the Ethics Committee of the Fifth Medical Center of Chinese People’s Liberation Army Hospital in Beijing, China. The main information corresponding to the hepatocellular carcinoma samples is provided in Supplementary Data Table 1. The samples were soaked in 4% paraformaldehyde (PFA) tissue fixative solution for 24 h before being embedded in paraffin and sectioned for experiments.

### Cell lines

All the human tumor cell lines used are listed in Supplementary Data Table 2. The cell lines used in this study were obtained from the Cell Resource Center, Peking Union Medical College, which is part of the National Science and Technology Infrastructure, the National Biomedical Cell-Line Resource, and stored in our laboratory.

### Tissue immunohistochemistry

To analyze the binding of OT3 or GTM peptides to human tumor tissues and the expression of NCL and protein-glutamine γ-glutamyltransferase K (TGM1) in different human tumor tissues, a tissue microarray (Biomax) containing 12 kinds of cancer tissues and paracarcinoma tissues was used. After deparaffinization, the tissue microarray was washed with phosphate-buffered saline (PBS) and incubated in 3% H_2_O_2_ for 15 min to inactivate endogenous peroxidase. Then, antigen retrieval and tissue permeabilization were performed with 0.25% Triton X-100. The nonspecific sites of the tissue microarray were blocked with normal goat serum, and then, the microarray was immunostained with an appropriate concentration of biotin-conjugated OT3 or GTM peptide and incubated at 4 °C overnight. After washing with 1× TBST, an HRP-conjugated streptavidin antibody was added, and the mixture was incubated at room temperature (RT) for 1 h.

For the NCL/TGM1 expression assay, mouse anti-human NCL antibody C23 (Santa Cruz Biotechnology, 1:500)/rabbit anti-human TGM1 antibody (ABclonal, 1:150) and HRP-conjugated anti-mouse IgG/HRP-conjugated anti-rabbit IgG (ZSGB-Bio) were added and incubated. The tissue microarray was washed, and 3’-diaminobenzidine (DAB) was added and incubated. Then, hematoxylin was used to rest the tissue, and the microarray was sealed with resin.

For analysis of the expression of NCL and 11 other protein ligands recognized by γδ T cells in liver puncture biopsy samples, paraffin sections of the samples were heated at 60 °C for 1 h, cooled naturally and then dewaxed. After being washed in PBS, the sections were heated in sodium citrate antigen repair solution, thus allowing the exposure of antigens. Eleven of the antibodies used in the assay were obtained from Santa Cruz Biotechnology [anti-HSP27, anti-HSP60, anti-HSP70, anti-HSP90, anti-ULBP1, anti-ULBP2, anti-ULBP3, anti-ULBP4, anti-MSH2, anti-NCL and anti-MICA/B antibodies (mouse)], and the rabbit anti-AGR2 antibody was obtained from Abcam. These antibodies were diluted in goat serum at a ratio of 1:250, added dropwise to the tissue and incubated overnight at 4 °C. Horseradish peroxidase (HRP)-conjugated anti-mouse IgG/HRP-conjugated anti-rabbit IgG (ZSGB-Bio) was used as described above for subsequent detection.

### Flow cytometric analysis

1. For analysis of the binding of the OT3 or GTM peptide to different human tumor cells, the tumor cells were washed and resuspended in FACS buffer (PBS with 1% BSA). Single-cell suspensions were incubated with 20 µg of biotin-conjugated OT3 or GTM peptide at 4 °C for 1 h. Then, the cells were stained for 30 min at 4 °C with an APC-conjugated streptavidin antibody (BioLegend).
2. For analysis of NCL expression in tumor cell lines, 1 μg of rabbit anti-human NCL (GeneTex) primary antibody was added, and the mixture was incubated for 30 min at RT. The secondary antibody, Alexa Fluor 647-conjugated goat anti-rabbit IgG (CST), was diluted to 1:500 and incubated for 10 min at RT. For analysis of hMSH2 expression in tumor cell lines, 1 μg of mouse anti-human MSH2 (Abcam) primary antibody was added, and the mixture was incubated for 1 h at 4 °C. FITC-conjugated goat anti-mouse IgG (BioLegend) at a 1:100 dilution was added, and the mixture was incubated for 30 min at 4 °C. For analysis of TGM1 expression, TGM1 polyclonal rabbit antibodies (ABclonal) were used as primary antibodies at a 1:100 dilution and incubated for 1 h. FITC-conjugated anti-rabbit IgG was then added at a 1:150 dilution and incubated for 30 min on ice. For the cell stress model, 293 T cells and tumor cells were treated with menadione (MedChemExpress) at certain concentrations and for certain durations. Menadione (20 μM) was used in the flow cytometric assay to assess the expression of NCL and TGM1. Tumor cells were exposed to 50 µM menadione for 15 min to simulate short-term, high-intensity stress.
3. To determine the total percentages of γδ T cells, CD69^+^ γδ T cells, CD25^+^ γδ T cells, Vδ1 T cells and Vδ2 T cells from amplified PBMCs, 1 μg of FITC-conjugated mouse anti-human γδ TCR (BioLegend), PE-conjugated mouse anti-human CD69 (BioLegend), APC-conjugated mouse anti-human CD25 (BioLegend), FITC-conjugated mouse anti-human Vδ1 TCR (Thermo Fisher) and PE-conjugated mouse anti-human Vδ2 TCR (BioLegend) were added, and the mixture was incubated for 30 min at 4  °C.
4. For the sorting assay, peripheral blood mononuclear cells (PBMCs) were 2-color stained with PE/Cy7-conjugated anti-CD3 and biotin-conjugated anti-human TCR γ/δ (BioLegend), with APC-conjugated streptavidin (BioLegend) as a secondary antibody. After stimulation for 2 and 4 days, the cells were subjected to 5-color staining with PE/Cy7-conjugated anti-CD3 (BioLegend), APC-conjugated anti-Vδ1 TCR (Miltenyi), APC/Cy7-conjugated anti-CD69 (BioLegend), Brilliant Violet 510-conjugated anti-TCR Vδ2 (BioLegend), and Percp/Cy5.5-conjugated anti-human CD25 (BioLegend) antibodies.
5. For analysis of stress-induced ligand expression, different tumor cells or cells treated with ionizing radiation at a dose of 5 Gy for 6, 24 or 48 h were collected for flow cytometric analysis. The cells were collected and counted, and 1×10^6^ cells in each group were incubated with 1% BSA and then stained with a primary antibody at 4 °C for half an hour. After washing, the cells were stained with a secondary antibody at 4 °C for half an hour and assessed by flow cytometry. Primary antibodies, including mouse anti-human ULBP3, ULBP4, MICA/B, HSP27, HSP60, HSP70, HSP90, hMSH2, and NCL; rabbit anti-human ULBP1 and ULBP2; and isotype mouse IgG1 and IgG2a antibodies, were obtained from Santa Cruz. FITC-conjugated anti-rabbit and FITC-conjugated anti-mouse secondary antibodies were purchased from BioLegend.
6. To evaluate the proliferation of T cells after lentiviral transduction, peripheral blood mononuclear cells (PBMCs) from healthy donors were harvested, adjusted to a concentration of 1×10^6^ cells/mL and subsequently stained with 1 μM CellTrace^TM^ Far Red per mL of cell suspension at 37 °C for 20 min in the dark. T-cell culture medium (RPMI-1640 with 10% FBS, 2 mM glutamine and 200 U/mL recombinant human IL-2) was added, and the mixture was incubated for 5 min. All the cells were harvested and resuspended in cold T-cell culture medium, and 1×10^6^ cells were seeded into well plates and activated with T-Cell TransAct (Miltenyi). After 24 h, activated T cells were infected with lentivirus at a multiplicity of infection (MOI) of 2. The proliferative capacity of Ab-γδ TCR- or chimeric antigen receptor (CAR)-T cells was determined by flow cytometry at 48 h after the removal of lentiviral stimulation.

Except for the sorting analysis, all flow cytometric analyses were performed with a BD Accuri C6 system, and all flow data were analyzed via C6 software. The sorted cell suspensions were analyzed via a FACS Cannto II system (Becton Dickinson). The acquired data were analyzed via Flow Jo software (Becton Dickinson).

### Microscale thermophoresis (MST)

The OT3 or GTM peptides were labeled with 1 μL of FITC-streptavidin (BioLegend) for 1.5 h on ice, and the fluorescence-labeled peptides were diluted to a concentration that yielded a fluorescence count between 200 and 1500. Next, stress-inducible proteins, including HSP27/60/70/90, hMSH2, MICA/B, and ULBP1/2/4/6 (Sino Biological, Inc.), were diluted to an appropriate concentration with buffer solution. Then, the protein and fluorescence-labeled peptide were mixed at a ratio of 1:1 for 5 min at RT, the samples were loaded into the capillaries, and the MST experiment was run with 40% MST power.

### Protein microarray

The microarray slides (CD) were initially blocked with 3% BSA-TBST at RT for 1 h. Serum was added, and the samples were incubated with the arrays for 1 h at RT. In the GTM-Fc protein microarray, GTM-Fc or Fc (Sino Biological, Inc.), as the primary antibody, was diluted, added, and the mixture was incubated at RT for 1 h. Then, a Cy3-conjugated goat anti-human IgG antibody (Bioss) was added, and the mixture was incubated for 1 h at RT. In the GTM-γδ TCR microarray, soluble Vγ4δ1 TCR with the GTM sequence in its CDR3δ sequence was used, followed by an anti-pan γδ TCR antibody and a Cy3-labeled goat anti-mouse IgG antibody (Bioss). A control experiment was carried out with a recombinant soluble Vγ9δ2 TCR. In the OT3 protein microarray, OT3 peptide or biotin, as the primary antibody, was diluted, added, and incubated at 4 °C for 12 h. Cy3-conjugated streptavidin (Bioss) was used to probe the primary antibodies bound to the slide at RT for 1 h.

For all the assays, a rabbit anti-GST monoclonal antibody (CST) was added and incubated at RT for 1 h. An Alexa Fluor 647-conjugated goat anti-rabbit IgG (CST) secondary antibody was added and incubated at RT for 1 h. Finally, the microarray slides were scanned with a GenePix 4000B fluorescent microarray scanner (GBI). The probe signals were acquired via GenePix Pro 6.0 software (Molecular Devices).

### Immunoprecipitation

The full-length NCL sequence was obtained from the American Type Tissue Culture Collection (ATCC) and cloned and inserted into CFUGW plasmids via Phusion DNA polymerase (Biolabs). The experimental procedures were performed according to the manufacturer’s instructions. The NCL plasmids were subsequently analyzed by sequencing. A vector with the correct sequence was transfected into 293 T cells via Lipofectamine 2000 (Invitrogen). Twenty microliters of anti-GFP-conjugated Sepharose beads (Biotool) were added to 293 T whole-cell lysates and incubated for 2 h at 4 °C with rotation. GTM-Fc (2 μg) and Fc (2 μg) were added to the system and incubated overnight at 4 °C with rotation. The pellets were washed six times with cell lysis buffer. Finally, the pellets were resuspended in 20 μL of 1× SDS sample buffer and heated to 95 ~ 100 °C for 2 ~ 5 min to remove the Sepharose beads. The samples were analyzed by Western blotting using an anti-human IgG antibody (ZSGB-Bio).

### Biolayer interferometry (BLI) assay

The BLI assay was performed with a ForteBio Octet Red 96 instrument to determine the affinity of GTM-TCR for FBXO2. In brief, 6×His-tagged GTM-TCR was immobilized onto Ni-NTA-coated biosensors and then dipped into a solution containing FBXO2 protein for 6 min, followed by dissociation for 5 min. Sensor grams of the concentration series were corrected with corresponding blank curves and fitted globally with Octet evaluation software via a 1:1 Langmuir model of binding. The affinity constant (KD) (mol/L) reflects the binding capacity.

### Confocal microscopy

1. Tumor cells, including HT-29 and BGC-823 cells, were placed on slides pretreated with polylysine overnight and fixed with 4% cold PFA. Fixed cells were permeabilized or not permeabilized and incubated with rabbit anti-human NCL (GeneTex) overnight at 4 °C. Alexa Fluor 647-conjugated goat anti-rabbit IgG antibody (CST) was then added, and the samples were incubated for 2 h at RT. Rabbit IgG (GeneTex) was used as an isotype control. The slides were examined via confocal microscopy (Zeiss).
2. For analysis of TGM1 expression, NCI-H520 cells or menadione-treated 293 T cells (for 15 min) and rabbit anti-human TGM1 (ABclonal, 1:100) and Alexa 488-conjugated anti-rabbit (A11008, 1:1000) antibodies were used following the same protocol.

### Menadione-induced cell stress model

NCI-H520 cells were treated with 0.5, 5, 10, 20, 40, or 80 μM menadione (MedChemExpress; untreated and DMSO-treated cells were used as controls) for 15 min and then collected for analysis of total protein expression. Western blotting with an anti-PAR monoclonal antibody (ENZO Life Sciences, 1:2000) was used to detect the characteristic modifications of PAR. Anti-β-actin (Sigma, 1:5000) was used for normalization. A Cell Counting Kit-8 assay (Dojindo Laboratories) was used to evaluate the effects of different treatment times and concentrations of menadione on the viability of both 293 T cells and the tumor cell line NCI-H520. The assay was conducted according to the manufacturer’s instructions.

### γδ T-cell expansion with recombinant proteins

Freshly isolated PBMCs from healthy donors were cultured in RPMI 1640 medium (Gibco BRL) supplemented with 10% FCS and IL-2 (200 U/mL) in 24-well culture plates supplemented with immobilized recombinant NCL (CUSABIO, 20 μg/mL). Plates with immobilized BSA (Sigma, 20 μg/mL) were used as a control. After 1 week of culture, the purity and subtype of the γδ T cells were analyzed via flow cytometry.

γδ T cells were sorted from freshly isolated PBMCs from healthy donors and cultured in RPMI 1640 medium (Gibco BRL) supplemented with 10% FBS and IL-2 (40 U/mL, Quanqi) in 96-well culture plates with immobilized recombinant NCL (CUSABIO, 20 μg/mL) and TGM1 (IPODIX, 20 μg/mL). The plates with immobilized medium, BSA (Sigma, 20 μg/mL), anti-Vδ1 TCR (Beckman Coulter, 1 μg/mL) and zoledronic acid (Kelun, 1 μmol/L) were used as controls. After 48 and 96 h of culture, the percentages of CD69+ and CD25^+^ Vδ1 T and Vδ2 T cells were analyzed via flow cytometry.

### Sorting γδ T cells from PBMCs

PBMCs were separated from peripheral blood via density gradient centrifugation via Ficoll‒Hypaque density gradient centrifugation fluid (MD Pacific). After cell counting and viability detection, the PBMCs were successively incubated with an anti-human TCRγ/δ antibody conjugated with biotin (BioLegend) and antibiotic GMP microbeads (Miltenyi, 170--076-709). Finally, γδ T cells were magnetically separated.

### Expansion of Vδ1 T cells from PBMCs

PBMCs were isolated from healthy donors via a Ficoll gradient prior to cryopreservation. Then, the PBMCs were thawed and plated onto an immobilized agonistic anti-Vδ1 TCR antibody for 2–4 days at a concentration of 1 × 10^6^ cells/mL. Human cytokines (100 ng/mL IL4, 70 ng/mL IFN-γ, 7 ng/mL IL-21, and 15 ng/mL IL-1β) were added to the medium in the first stage for 8 days. The cells were incubated at 37 °C and 5% CO2. In the second stage, fresh medium supplemented with cytokines (70 ng/mL IL-15 and 30 ng/mL IFN-γ) was replaced every 5 to 6 days until day 21. The percentage of Vδ1 TCRs was analyzed via flow cytometry.

### Enzyme-linked immunosorbent assay (ELISA)

Vδ1 T cells (purity over 90%) that were expanded from PBMCs with an anti-Vδ1 TCR monoclonal antibody (Immunotech) were cultured in medium without IL-2 for 24 h before stimulation with NCL (CUSABIO, 50 μg/mL) or BSA (Sigma, 50 μg/mL) for 36 h and PMA/ION for 4 h. Human IFN-γ and IL-2 ELISA kits (4 A Biotech) were used to measure the concentrations of IFN-γ and IL-2 in the cell supernatants. The experimental procedure followed the manufacturer’s instructions.

### Lactate dehydrogenase (LDH) cytotoxicity assay

1. A systematic cytotoxicity assay was performed to assess the effects of human γδ T cells on 50 human tumor cell lines. Different tumor cell lines (5 × 10^4^ per well) were plated in round-bottom plates (96 wells). Human γδ T cells expanded from healthy PBMCs by incubation with immobilized anti-γδ TCR antibodies for 10 days (purity > 90%) were added at an effector:target (E:T) ratio of 10:1 for 6 h. All the groups were assayed in triplicate. The cytotoxicities of the γδ T cells against different target tumor cells are shown relative to the cytotoxicities of the γδ T cells against Daudi cells to avoid differences in the cytotoxicity of different effector γδ T cells, which were expanded from different healthy donors.
2. To assess Vδ1 T-cell cytotoxicity against tumor cells, HepG2, MDA-MB-231, and OVCAR-8 cells were added to 96-well plates at a density of 3 × 10^4^ cells/well and incubated with anti-NCL or isotype antibodies for 6 h at 37 °C. Vδ1 T cells were added to the plates as effector cells at an E:T ratio of 10:1, and each condition was assayed in triplicate.
3. To analyze the cytotoxicity of γδ T cells to menadione-treated tumor cells, HepG2, NCI-H520, and OVCAR-8 cells were treated with 50 µM menadione for 15 min (untreated cells were used as controls) and then used as target cells. γδ T cells (E:T = 5:1) were added and incubated for 6 h at 37 °C.
4. For radiation induction, Daudi cells were exposed to ionizing radiation at a dose of 5 Gy, with untreated cells serving as controls. The irradiated Daudi cells were then cocultured with γδ T cells (1 × 10^5^) for a cytotoxicity assay at an effector-to-target (E:T) ratio of 10:1 in a final volume of 100 µL per well at various time points following radiation exposure.

We assessed cytotoxicity via an LDH assay via the CytoTox 96 Nonradioactive Cytotoxicity Assay Reagent Kit (Promega). The cytotoxicity assay was conducted according to the manufacturer’s instructions.

### Real-time cellular analysis (RTCA)

The medium of the tumor cell lines was adjusted to the proper concentrations, and 1 × 10^4^ cells were seeded into well plates and monitored via RTCA (xCELLigence S16, ACEC Biosciences). The tumor cells were cultured for 24 h to allow cell adherence before the effector cells were added. The number of adjacent tumor cells (living target cells) was continuously recorded. Cytotoxicity was quantified by subtracting the percentage of viable target cells cultured alone from that of target cells cocultured with effector cells.

For radiation induction, adjacent tumor cells were treated with ionizing radiation at a dose of 5 Gy (untreated cells were used as controls). γδ T cells (1 × 10^5^) were added at an E:T ratio of 10:1 in a final volume of 100 µL/well. Tumor cells were cultured alone or cocultured with γδ T cells for another 32 h.

For the in vitro cytotoxicity assay, MOCK-αβ T, hMSH2-Ab-γδ TCR-αβ T, NCL-Ab-γδ TCR-αβ T or hMSH2-CAR-αβ T cells (5×10^4^ ZsGreen^+^ cells) were added at an E:T ratio of 5:1 in a final volume of 200 µL/well. Tumor cells were cultured alone or cocultured with different effector cells for another 24 h.

### γδ CAR-T-cell construction

The variable region of the TCRγ9 and TCRδ2 chains with the tumor antigen-specific CDR3 region OT3, which was sequenced from γδ T cells infiltrating ovarian epithelial carcinoma tissues, was directly synthesized by the TSINGKE Company. The sequences were subsequently cloned and inserted into an MSCV retro vector containing the sequence CD8-CD28BBZ. The amplified γ9δ2-CD8-CD28BBZ sequence was then cloned and inserted into a cFUGW lentiviral vector containing a Flag tag at the N-terminus and GFP. The γδ CAR vector was transfected into 293 T cells in a 10-cm dish with the psPAX2 packaging plasmid and PMD2. G envelope plasmid following the instructions for jetPRIME transfection reagent (Polyplus). Then, the 0.45-µm filtered virus supernatant was concentrated by ultrafiltration and resuspended in RPMI-1640 medium.

PBMCs from healthy donors were isolated via density gradient centrifugation and activated with 1 µg/mL human CD3 NALE HIT3a and 5 µg/mL human CD28 NALE CD28.2 antibodies (BD) for 24 h. T cells were then transduced with γδ CAR lentivirus at an MOI of 2 and centrifuged at 1800 rpm for 60 min at 30 °C. After 20–24 h, the activated antibodies and lentivirus were removed via centrifugation, the T cells were cultured for another 48 h in RPMI-1640 medium supplemented with 10% FBS, 2 mM glutamine and 200 U/mL recombinant human IL-2, and the GFP signal corresponding to the γδ CAR-positive T cells was evaluated via flow cytometry. All the results were obtained from samples from no fewer than 3 donors.

### Generation of Ab γδ TCR-T cells and CAR-T cells targeting hMSH2 and NCL

The sequences of the variable regions of the anti-hMSH2 and anti-NCL antibodies were derived from CN 110218251 A and WO 2017/156032 A1, respectively. The sequences of the conserved regions of the human TCR γ and δ chains were obtained from IMGT. The CAR is a third-generation CAR containing the CD28/41BB/CD3ζ domains. After sequence synthesis, Ab-γδ TCRs and CARs were cloned and inserted into the pLVX lentiviral vector via homologous recombination for delivery into T cells. PBMCs from healthy donors were isolated via density gradient centrifugation and activated with T-Cell TransAct (Miltenyi). After 24 h, activated T cells were infected with lentivirus at an MOI of 2 and cultured in RPMI-1640 medium supplemented with 10% FBS, 2 mM glutamine and 200 U/mL recombinant human IL-2. The transduction efficacy of Ab-γδ TCRs and CARs in T cells was determined by flow cytometry. All results with the Ab-γδ TCR and CAR were obtained with samples from no fewer than 3 donors.

### Western blotting

For colorectal carcinoma cell lines, cells were collected for analysis of FBXO2 expression via anti-FBX02 antibodies (Proteintech).

After 24 h of plasmid transfection, the 293 T cells were collected for analysis of total TCR δ (Santa Cruz), CD3Zeta (Abcam), and β-actin (Sigma) protein expression.

For T cells, after 4 days of expansion, lentivirus-infected T cells were collected for analysis of total protein expression. Antibodies recognizing CD3Zeta (Abcam), Flag (Sigma), β-actin (Applygen) and GFP (Chemotek) were added and incubated overnight at 4 °C. All the primary antibodies were detected via HRP-conjugated anti-mouse or anti-rabbit antibodies (ZSGB-Bio), which were added and incubated at RT for 2 h. A chemiluminescence solution (Thermo Fisher) was used to detect the expression of the proteins.

### Verification of Ab-γδ TCR-αβ T-cell specificity

The NCI-H520 and A549 cell lines were transfected with hMSH2-GFP and NCL-GFP (Addgene) plasmids, respectively. After 24 h, the expression of GFP was observed via fluorescence microscopy. A total of 1 × 10^4^ tumor cells (NCI-H520/NCI-H520-hMSH2) were seeded into well plates, and hMSH2-Ab-γδ TCR-αβ T cells were added to achieve an E:T ratio of 5:1 in a final volume of 200 µL/well. The results were monitored by RTCA (xCELLigence S16, ACEC Biosciences) for 24 h as described above. For NCL-Ab-γδ TCR-αβ T cells, A549/A549-mock/A549-NCL cells were used as target cells, and untreated αβ T, MOCK-αβ T, or NCL-Ab-γδ TCR-αβ T cells were employed as effector cells at an E:T ratio of 5:1. We evaluated cytotoxicity via an LDH assay, as described above.

### Animal model and treatment

Animal experiments were performed according to the guidelines of the Animal Use Committee of the Experimental Animal Center, Institute of Basic Medical Sciences, Chinese Academy of Medical Sciences.

For radiotherapy, 2 × 10^6^ NCI-H520 lung cancer tumor cells were subcutaneously engrafted into the backs of BALB/c nude mice, and 32 mice were randomly divided into four groups when the tumor diameter reached 5 ~ 9 mm. The mice were then given ionizing radiation at a dose of 2 Gy at the local tumor site once every four days (two total treatments) and injected with 1 × 10^7^ γδ T cells near the tumors 24 h after radiation.

For γδ CAR-T cells, OVCAR-8 cells were transfected with luciferase-IRES-GFP viral supernatant, and the resulting cell clones were sorted via flow cytometry on the basis of the expression of GFP. Female 6 ~ 8-week-old NCG mice were obtained from the Model Animal Research Center of Nanjing University. Stable OVCAR-8 cells labeled with luciferase were subcutaneously injected (3 × 10^6^ cells per mouse) in the right flank. On days 7, 10, 14, and 18 after tumor injection, GFP-αβ T cells or γδ CAR-αβ T cells (1 × 10^7^ cells per mouse) were intratumorally injected. Then, luciferase expression was detected by using D-Luciferin Firefly in vivo Imaging Reagents (PerkinElmer), and the results were analyzed via live imaging software. The animal experiments were performed according to the guidelines of the Animal Use Committee of the Experimental Animal Center, Institute of Basic Medical Sciences, Chinese Academy of Medical Sciences.

For experiments with Ab-γδ TCR-T and CAR-T cells, female 6 ~ 8-week-old B-NDG mice (10 for each group) were obtained from Biocytogen Pharmaceuticals (Beijing). SW480 cells and HepG2 cells were obtained from the Cell Center of the Chinese Academy of Medical Sciences and injected subcutaneously (5 × 10^6^ cells per mouse) into the right flank. For the SW480 tumor-bearing mouse model, MOCK-αβ T or hMSH2-Ab-γδ TCR-αβ T cells (2 × 10^6^ cells per mouse) were intratumorally injected 4 times on days 9, 13, 17, 21 and 25 (with PBS as a control), and hMSH2-CAR-αβ T cells (1 × 10^7^ cells per mouse) were injected only once, on day 9. For the HepG2 tumor-bearing mouse model, MOCK-αβ T or NCL-Ab-γδ TCR-αβ T cells (1× 10^6^ cells per mouse) were intratumorally injected 4 times on days 13, 16, 19 and 22 (with PBS as a control), and NCL-CAR-αβ T cells (5 × 10^6^ cells per mouse) were intratumorally injected only once, on day 13 after tumor injection. Tumors were measured with a Vernier caliper every 3 or 4 days, and the volume was calculated according to the following equation: tumor volume (mm^3^) = maximal length (mm) ×perpendicular width^2^ (mm^2^)/2. When the tumor volume exceeded 2000 mm^3^, the mice were sacrificed, and the survival time was recorded.

### Statistical analyses

Statistical analyses of flow cytometric data, γδ T-cell expansion data and in vitro cytotoxicity assay data were performed via appropriate statistical tests, including paired or unpaired two-tailed *t* tests with Welch’s correction as needed and one-way analysis of variance (ANOVA) with multiple comparisons (SPSS 13.0 software).

## Supplementary information

supplemental data

Unprocessed original images of gels and western blots

## References

[1] Chien YH, Meyer C, Bonneville M. γδ T cells: first line of defense and beyond. Annu Rev Immunol. 2014;32:121–55. 10.1146/annurev-immunol-032713-120216 24387714 10.1146/annurev-immunol-032713-120216

[2] Vantourout P, Hayday A. Six-of-the-best: unique contributions of γδ T cells to immunology. Nat Rev Immunol. 2013;13:88–100. 10.1038/nri3384 23348415 10.1038/nri3384 PMC3951794

[3] Godfrey DI, Uldrich AP, McCluskey J, Rossjohn J, Moody DB. The burgeoning family of unconventional T cells. Nat Immunol. 2015;16:1114–23. 10.1038/ni.3298 26482978 10.1038/ni.3298

[4] Adams EJ, Chien YH, Garcia KC. Structure of a gammadelta T-cell receptor in complex with the nonclassical MHC T22. Science. 2005;308:227–31. 10.1126/science.1106885 15821084 10.1126/science.1106885

[5] Vavassori S, Kumar A, Wan GS, Ramanjaneyulu GS, Cavallari M, El Daker S, et al. Butyrophilin 3A1 binds phosphorylated antigens and stimulates human γδ T cells. Nat Immunol. 2013;14:908–16. 10.1038/ni.2665 23872678 10.1038/ni.2665

[6] Yuan L, Ma X, Yang Y, Qu Y, Li X, Zhu X, et al. Phosphoantigens glue butyrophilin 3A1 and 2A1 to activate Vγ9Vδ2 T cells. Nature. 2023;621:840–8. 10.1038/s41586-023-06525-3 37674084 10.1038/s41586-023-06525-3 PMC10533412

[7] Wu J, Groh V, Spies T. T-cell antigen receptor engagement and specificity in the recognition of stress-inducible MHC class I-related chains by human epithelial gamma delta T cells. J Immunol. 2002;169:1236–40. 10.4049/jimmunol.169.3.1236 12133944 10.4049/jimmunol.169.3.1236

[8] Ribot JC, Lopes N, Silva-Santos B. γδ T cells in tissue physiology and surveillance. Nat Rev Immunol. 2021;21:221–32. 10.1038/s41577-020-00452-4 33057185 10.1038/s41577-020-00452-4

[9] Willcox CR, Mohammed F, Willcox BE. The distinct MHC-unrestricted immunobiology of innate-like and adaptive-like human γδ T-cell subsets-Nature’s CAR-T cells. Immunol Rev. 2020;298:25–46. 10.1111/imr.12928 33084045 10.1111/imr.12928

[10] Allison TJ, Garboczi DN. Structure of gammadelta T-cell receptors and their recognition of nonpeptide antigens. Mol Immunol. 2002;38:1051–61. 10.1016/s0161-5890(02)00034-2 11955597 10.1016/s0161-5890(02)00034-2

[11] Das H, Wang L, Kamath A, Bukowski JF. Vgamma2Vdelta2 T-cell receptor-mediated recognition of aminobisphosphonates. Blood. 2001;98:1616–8. 10.1182/blood.v98.5.1616 11520816 10.1182/blood.v98.5.1616

[12] Uldrich AP, Le Nours J, Pellicci DG, Gherardin NA, McPherson KG, Lim RT, et al. CD1d-lipid antigen recognition by the γδ TCR. Nat Immunol. 2013;14:1137–45. 10.1038/ni.2713 24076636 10.1038/ni.2713

[13] Alves I, Santos-Pereira B, de la Cruz N, Campar A, Pinto V, Rodrigues PM, et al. Host-derived mannose glycans trigger a pathogenic γδ T-cell/IL-17a axis in autoimmunity. Sci Transl Med. 2023;15:eabo1930. 10.1126/scitranslmed.abo1930 36921032 10.1126/scitranslmed.abo1930

[14] Crowley MP, Reich Z, Mavaddat N, Altman JD, Chien Y. The recognition of the nonclassical major histocompatibility complex (MHC) class I molecule, T10, by the gammadelta T cell, G8. J Exp Med. 1997;185:1223–30. 10.1084/jem.185.7.1223 9104809 10.1084/jem.185.7.1223 PMC2196254

[15] Xu C, Zhang H, Hu H, He H, Wang Z, Xu Y, et al. Gammadelta T cells recognize tumor cells via CDR3delta region. Mol Immunol. 2007;44:302–10. 10.1016/j.molimm.2006.03.010 16650897 10.1016/j.molimm.2006.03.010

[16] Jiang Y, Guo Y, Xi X, Cui L, He W. Flanking V and J sequences of complementary determining region 3 of T-cell receptor (TCR) δ1 (CDR3δ1) determine the structure and function of TCRγ4δ1. J Biol Chem. 2011;286:25611–9. 10.1074/jbc.M111.239624 21606499 10.1074/jbc.M111.239624 PMC3138283

[17] Chen H, He X, Wang Z, Wu D, Zhang H, Xu C, et al. Identification of human T-cell receptor gammadelta-recognized epitopes/proteins via CDR3delta peptide-based immunobiochemical strategy. J Biol Chem. 2008;283:12528–37. 10.1074/jbc.M708067200 18321859 10.1074/jbc.M708067200

[18] Xi X, Guo Y, Chen H, Xu C, Zhang H, Hu H, et al. Antigen specificity of gammadelta T cells depends primarily on the flanking sequences of CDR3delta. J Biol Chem. 2009;284:27449–55. 10.1074/jbc.M109.011684 19666468 10.1074/jbc.M109.011684 PMC2785674

[19] He K, You H, Li Y, Cui L, Zhang J, He W. TCRγ4δ1-engineered αβT cells exhibit effective antitumor activity. Mol Med. 2016;22:519–29. 10.2119/molmed.2016.00023 27463149 10.2119/molmed.2016.00023 PMC5082300

[20] Zhao H, Xi X, Cui L, He W. CDR3δ -grafted γ9δ2T cells mediate effective antitumor reactivity. Cell Mol Immunol. 2012;9:147–54. 10.1038/cmi.2011.28 21909128 10.1038/cmi.2011.28 PMC4002801

[21] Kong Y, Cao W, Xi X, Ma C, Cui L, He W. The NKG2D ligand ULBP4 binds to TCRgamma9/delta2 and induces cytotoxicity to tumor cells through both TCRgammadelta and NKG2D. Blood. 2009;114:310–7. 10.1182/blood-2008-12-196287 19436053 10.1182/blood-2008-12-196287

[22] Zhao J, Huang J, Chen H, Cui L, He W. Vdelta1 T-cell receptor binds specifically to MHC I chain related A: molecular and biochemical evidence. Biochem Biophys Res Commun. 2006;339:232–40. 10.1016/j.bbrc.2005.10.198 16297874 10.1016/j.bbrc.2005.10.198

[23] Zhang H, Hu H, Jiang X, He H, Cui L, He W. Membrane HSP70: the molecule triggering gammadelta T cells in the early stage of tumorigenesis. Immunol Invest. 2005;34:453–68. 10.1080/08820130500265349 16302688 10.1080/08820130500265349

[24] Yu JJ, Zhou DD, Yang XX, Cui B, Tan FW, Wang J, et al. TRIB3-EGFR interaction promotes lung cancer progression and defines a therapeutic target. Nat Commun. 2020;11:3660. 10.1038/s41467-020-17385-0 32694521 10.1038/s41467-020-17385-0 PMC7374170

[25] Zhang X, Xiao S, Rameau RD, Devany E, Nadeem Z, Caglar E, et al. Nucleolin phosphorylation regulates PARN deadenylase activity during cellular stress response. RNA Biol. 2018;15:251–60. 10.1080/15476286.2017.1408764 29168431 10.1080/15476286.2017.1408764 PMC5798948

[26] Shi H, Huang Y, Zhou H, Song X, Yuan S, Fu Y, et al. Nucleolin is a receptor that mediates antiangiogenic and antitumor activity of endostatin. Blood. 2007;110:2899–906. 10.1182/blood-2007-01-064428 17615292 10.1182/blood-2007-01-064428

[27] Steinert PM, Kim SY, Chung SI, Marekov LN. The transglutaminase 1 enzyme is variably acylated by myristate and palmitate during differentiation in epidermal keratinocytes. J Biol Chem. 1996;271:26242–50. 10.1074/jbc.271.42.26242 8824274 10.1074/jbc.271.42.26242

[28] Kim W, Lee S, Seo D, Kim D, Kim K, Kim E, et al. (2019) Cellular stress responses in radiotherapy. Cells 8. 10.3390/cells8091105 10.3390/cells8091105 PMC6769573 31540530

[29] Lew AM, Pardoll DM, Maloy WL, Fowlkes BJ, Kruisbeek A, Cheng SF, et al. Characterization of T-cell receptor gamma chain expression in a subset of murine thymocytes. Science. 1986;234:1401–5. 10.1126/science.3787252 3787252 10.1126/science.3787252

[30] Haas W, Pereira P, Tonegawa S. Gamma/delta cells. Annu Rev Immunol. 1993;11:637–85. 10.1146/annurev.iy.11.040193.003225 8476575 10.1146/annurev.iy.11.040193.003225

[31] Lee D, Rosenthal CJ, Penn NE, Dunn ZS, Zhou Y, Yang L. Human γδ T-cell subsets and their clinical applications for cancer immunotherapy. Cancers 2022;14. 10.3390/cancers14123005 10.3390/cancers14123005 PMC9221220 35740670

[32] Lafaille JJ, DeCloux A, Bonneville M, Takagaki Y, Tonegawa S. Junctional sequences of T-cell receptor gamma delta genes: implications for gamma delta T-cell lineages and for a novel intermediate of V-(D)-J joining. Cell. 1989;59:859–70. 10.1016/0092-8674(89)90609-0 2590942 10.1016/0092-8674(89)90609-0

[33] Wilson IA, Stanfield RL. Unraveling the mysteries of gammadelta T-cell recognition. Nat Immunol. 2001;2:579–81. 10.1038/89718 11429538 10.1038/89718

[34] Mensurado S, Blanco-Domínguez R, Silva-Santos B. The emerging roles of γδ T cells in cancer immunotherapy. Nat Rev Clin Oncol. 2023;20:178–91. 10.1038/s41571-022-00722-1 36624304 10.1038/s41571-022-00722-1

[35] Ding Y, Song N, Liu C, He T, Zhuo W, He X, et al. Heat shock cognate 70 regulates the translocation and angiogenic function of nucleolin. Arterioscler Thromb Vasc Biol. 2012;32:e126–34. 10.1161/atvbaha.112.247502 22743058 10.1161/ATVBAHA.112.247502

[36] Wu DM, Zhang P, Liu RY, Sang YX, Zhou C, Xu GC, et al. Phosphorylation and changes in the distribution of nucleolin promote tumor metastasis via the PI3K/Akt pathway in colorectal carcinoma. FEBS Lett. 2014;588:1921–9. 10.1016/j.febslet.2014.03.047 24713430 10.1016/j.febslet.2014.03.047

