# Supplementary material for: γδ T-cell autoresponses to ectopic membrane proteins: a new type of pattern recognition
Source: Cell Mol Immunol. 2025 Feb 13;22(4):356–70. doi: 10.1038/s41423-025-01258-x (PMC11955531; doi:10.1038/s41423-025-01258-x)
Supplement: Supplementary file 1 — supplemental data [file 41423_2025_1258_MOESM1_ESM.docx]

**Supplementary data**

Supplementary data table 1. Main information for hepatocellular carcinoma samples.

| Number | Sex | Age | Type | Child‒Pugh score | BCLC stage |
| --- | --- | --- | --- | --- | --- |
| YT02-001 | Male | 65 | HBV-cirrhosis HCC (moderately differentiated) | B-7 | A3 |
| YT02-004 | Male | 67 | HBV-cirrhosis HCC | A-5 | A3 |
| YT02-006 | Male | 54 | HBV-cirrhosis HCC | A-6 | A3 |
| YT02-007 | Male | 65 | HBV-cirrhosis HCC | A-5 | A3 |
| YT02-008 | Male | 68 | HBV-cirrhosis HCC (moderately differentiated) | B-8 | A |
| YT02-009 | Male | 61 | HBV-cirrhosis HCC (moderately differentiated) | A-5 | B |

Supplementary data table 2. Human tumor cell lines.

| Tumor type | Cell lines | Tumor type | Cell lines |
| --- | --- | --- | --- |
| Esophageal carcinoma | EC-109 | Choriocarcinoma | JEG-3/Vp16 |
| Gastric carcinoma | MGC-803 | Endometrial adenocarcinoma | HEC-1B |
|  | HGC-27 | Ovarian cancer | ES-2 |
|  | BGC-823 |  | HO-8910 |
| Hepatocellular carcinoma | Hep3B |  | OVCAR-8 |
|  | HepG2 | Cervical cancer | CASK-1 |
|  | SMMC-7721 |  | HeLa |
| Pancreatic carcinoma | PANC-1 | Breast cancer | MDA-MB-231 |
| Colorectal carcinoma | CACO-2 | Melanoma | A375 |
|  | HT-29 | Neuroglioma | SNB19 |
|  | HR8348 |  | SF-268 |
|  | HCT116 |  | U251 |
|  | SW-480 | Myeloma | RPMI-8226 |
|  | SW-620 |  | SH-SY5Y |
|  | LoVo | B lymphoma | Raji |
| Nasopharyngeal carcinoma | CNE-1 |  | Daudi |
| Laryngocarcinoma | HEp-2 |  | RAMOS |
| Lung cancer | A549 |  | L428 |
|  | GLC-82 |  | SU-DHL-6 |
|  | NCI-H446 |  | Nalm-6 |
|  | NCI-H520 | T lymphoma | Jurkat-E6-1 |
|  | NCI-H2228 |  | huT78 |
|  | H69AR | Myeloid leukemia | U937 |
| Renal carcinoma | G401 |  | K562 |
|  | A498 |  |  |
|  | HK-2 |  |  |

Supplementary data table 3. Information about candidate proteins that bind to GTM and OT3.

| No | Abbreviation | Full name | Subcellular location | Function | Probe |
| --- | --- | --- | --- | --- | --- |
| 1 | ANKRD22 | ankyrin repeat domain 22 | Nucleus | Transcription factor, promotes the expression of E2F1, participates in the development of non-small cell carcinoma and esophageal cancer | GTM |
| 2 | CPNE4 | copine IV | Cytoplasm, nucleus | Phospholipids binding, involved in calcium-dependent signal transduction | GTM |
| 3 | CPNE2 | copine II | Plasma membrane, cytoplasm, nucleus | Phospholipids binding, involved in calcium-dependent signal transduction | GTM |
| 4 | FBXO2 | F-box protein 2 | Cytoplasm | Constitutes an ubiquitin-protein ligase complex, involved in ubiquitin-dependent phosphorylation | GTM |
| 5 | STARD10 | StAR-related lipid transfer domain containing 10 | Cytoplasm, nucleus | Phospholipids binding and transportation, regulation of cell proliferation and tumor genesis | GTM |
| 6 | VCX3A | variable charge, X-linked 3A | Nucleus | Cancer testis antigen | GTM |
| 7 | NCL | Nucleolin | Plasma membrane, nucleus | Ribosome transcription, assembly and maturation, transcriptional regulation of gene expression, receptor for ligands, such as growth factors and chemokines | GTM |
| 8 | ANAPC15 | Anaphase-promoting complex subunit 15 | Cytosol, nucleoplasm | Component of the anaphase promoting complex/cyclosome (APC/C), a cell cycle-regulated E3 ubiquitin ligase that controls progression through mitosis and the G1 phase of the cell cycle. | GTM |
| 9 | SLC7A6OS | Probable RNA polymerase II nuclear localization protein | Cytoplasm, nucleus | Directs RNA polymerase II nuclear import | GTM |
| 10 | YARS | Tyrosine-tRNA ligase, cytoplasmic | Cytoplasm, nucleus | Acts as a positive regulator of poly-ADP-ribosylation | GTM |
| 11 | HIST1H1B | Histone H1b | Nucleus | Acts as a regulator of individual gene transcription through chromatin remodeling, nucleosome spacing and DNA methylation | GTM |
| 12 | AIDA | Axin interactor, dorsalization-associated protein | Cytoplasm, membrane | Inhibits axin-mediated JNK activation by binding axin and disrupting axin homodimerization | GTM |
| 13 | VEGF | Vascular endothelial growth factor | Cytoplasm, nucleus | Participates in the induction of key genes involved in the response to hypoxia and in the induction of angiogenesis | GTM |
| 14 | TGM1 | Protein-glutamine gamma-glutamyltransferase K | Membrane; lipid-anchor | Catalyzes the cross-linking of proteins and the conjugation of polyamines to proteins; involved in cell proliferation | OT3 |
| 15 | SMAD7 | Mothers against decapentaplegic homolog 7 | Nucleus; cytoplasm | Antagonist of signaling by TGF-beta (transforming growth factor) type 1 receptor superfamily members | OT3 |
| 16 | ACADVL | Very long-chain specific acyl-CoA dehydrogenase | Membrane, mitochondrion inner membrane | Involved in the mitochondrial fatty acid beta-oxidation pathway, active toward esters of long-chain and very long-chain fatty acids | OT3 |

Supplementary data table 4. Comparison of the relative cytotoxicity of γδ T cells to a variety of human tumor cell lines.

|  | Number | Cancer type | Tumor cell lines | Ratio | E:T=10：1 |
| --- | --- | --- | --- | --- | --- |
| Solid | 1 | Esophageal carcinoma | EC-109 | 0.4066 | + |
| Tumor | 2 | Gastric carcinoma | MGC-803 | 0.8532 | ++ |
|  |  |  | HGC-27 | 1.0350 | +++ |
|  |  |  | BGC-823 | 0.6846 | ++ |
|  | 3 | Hepatocellular carcinoma | Hep-3B | 1.0136 | +++ |
|  |  |  | HepG2 | 3.3991 | +++++ |
|  |  |  | SMMC-7721 | 0.0524 | - |
|  | 4 | Pancreatic carcinoma | PANC-1 | 0.9655 | ++ |
|  | 5 | Colorectal carcinoma | CACO-2 | 0.2277 | + |
|  |  |  | HT-29 | 0.0612 | - |
|  |  |  | HR8348 | 1.5126 | +++ |
|  |  |  | HCT116 | 0.4044 | + |
|  |  |  | SW-480 | 0.4309 | + |
|  |  |  | SW-620 | 0.9529 | ++ |
|  |  |  | LoVo | 0.1367 | - |
|  | 6 | Nasopharyngeal carcinoma | CNE-1 | 0.1495 | + |
|  | 7 | Laryngocarcinoma | HEp-2 | 0.0618 | - |
|  | 8 | Lung cancer | A549 | 0.1491 | - |
|  |  |  | GLC-82 | 0.3582 | + |
|  |  |  | NCI-H446 | 0.4280 | + |
|  |  |  | NCI-H520 | 0.0467 | - |
|  |  |  | NCI-H2228 | 0.2862 | + |
|  |  |  | H69AR | 0.1712 | - |
|  | 9 | Renal carcinoma | G401 | 0.6498 | ++ |
|  |  |  | A498 | 0.9665 | ++ |
|  |  |  | HK-2 | 0.2991 | + |
|  | 10 | Choriocarcinoma | JEG-3/Vp16 | 2.2076 | ++++ |
|  | 11 | Endometrial carcinoma | HEC-1B | 0.4148 | + |
|  | 12 | Ovarian cancer | ES-2 | 0.1028 | - |
|  |  |  | HO-8910 | 0.4741 | + |
|  |  |  | OVCAR-8 | 0.5168 | ++ |
|  | 13 | Cervical carcinoma | CASK-1 | 1.3131 | +++ |
|  |  |  | HeLa | 0.0150 | - |
|  | 14 | Breast cancer | MDA-MB-231 | 0.3252 | + |
|  | 15 | Melanoma | A375 | 0.6083 | ++ |
|  | 16 | Neuroglioma | SNB19 | 0.2628 | + |
|  |  |  | SF-268 | 0.5704 | ++ |
|  |  |  | U251 | 0.1728 | - |
| Hematological tumors | 17 | Myeloma | RPMI-8226 | 4.0189 | ++++++ |
|  |  |  | SH-SY5Y | 0.4762 | + |
|  | 18 | B lymphoma | Raji | 0.1802 | - |
|  |  |  | Daudi | 1 | +++ |
|  |  |  | RAMOS | 0.5249 | ++ |
|  |  |  | L428 | 0.6213 | ++ |
|  |  |  | SU-DHL-6 | 0.8898 | ++ |
|  |  |  | Nalm-6 | 0.0425 | - |
|  | 19 | T lymphoma | Jurkat-E6-1 | 1.1756 | +++ |
|  |  |  | huT78 | 1.4862 | +++ |
|  | 20 | Myeloid lymphoma | U937 | 0.7619 | ++ |
|  |  |  | K562 | 1.1238 | +++ |

Supplementary Data Fig 1. Construction of OT3- and GTM-γδ CAR-T cells.


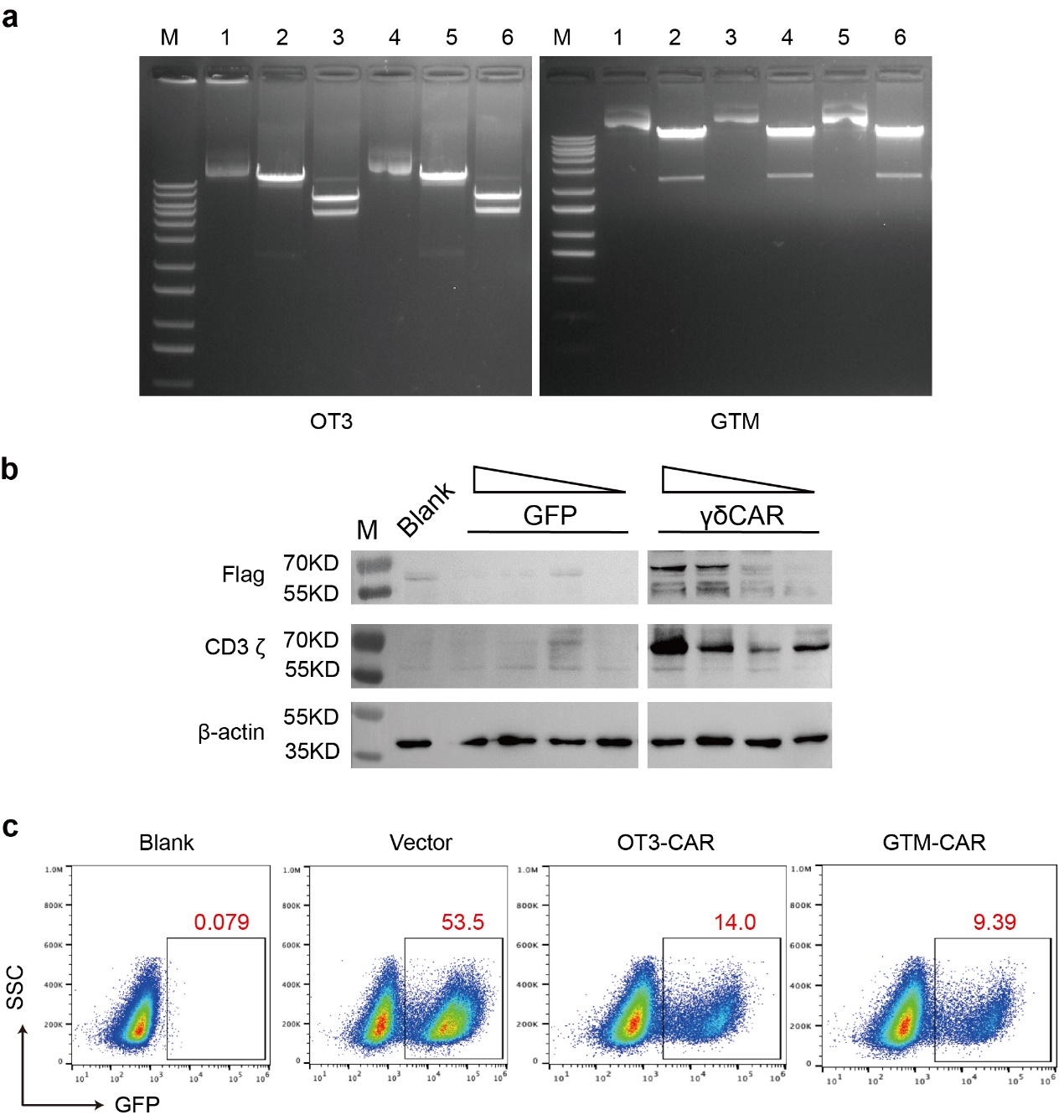


a. Enzyme digestion assays were applied to identify the correct insertion of the recombinant γδ CARs. M, DNA marker; OT3 CAR (left): lane 1 and 4, vector undigested; lane 2 and 5, vector digested with AvrII and RsrII; lane 3 and 6, vector digested with NheI and NdeI. GTM CAR (right): lane 1, 3 and 5, vector undigested; lane 2, 4 and 6, vector digested with AvrII and RsrII. b. Western blotting was performed to evaluate the expression of two different γδ CARs in 293T cells by using anti-CD3ζ and anti-flag antibodies. c. Flow cytometry was performed to evaluate the expression of two γδ CARs in αβT cells after lentiviral infection by measuring the GFP fluorescence signal. Approximately 10% of αβT cells expressed OT3-CAR and GTM-CAR, while the vector control infection efficiency was 50%.

Supplementary Data Fig 2. Validation of the direct binding of NCL to GTM probe.


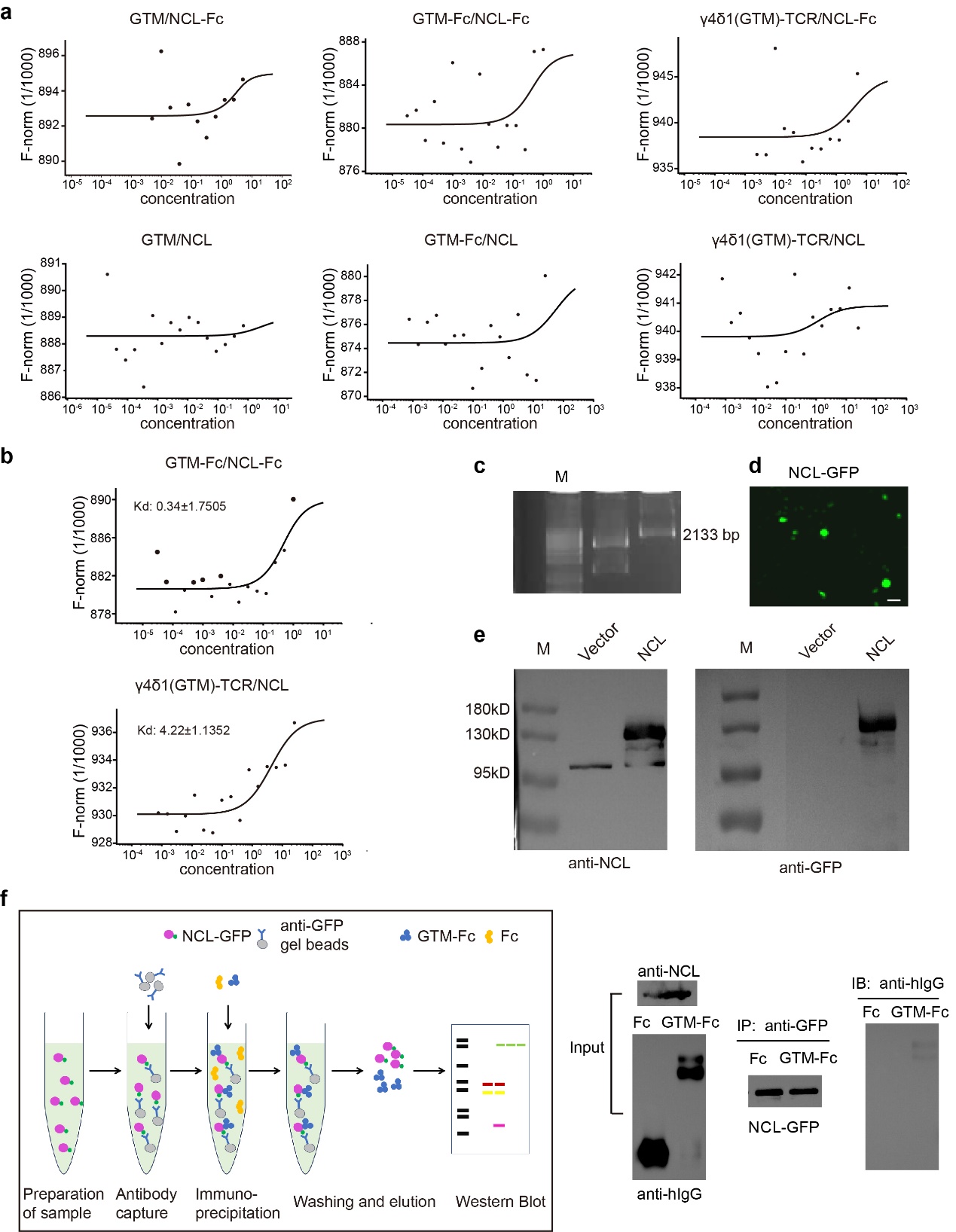


a. MST analysis of the binding activities of the GTM peptide, GTM-Fc, and γ4δ1(GTM)-TCR to NCL or NCL-Fc. To analyze the direct binding activities of GTM to NCL in MST, we used 2 kinds of recombinant NCL protein. NCL is a recombinant N-terminal 6 x his-tagged N-domain and partial central domain from 2-482 aa of NCL which is expressed by yeast (CUSABIO, China), and NCL-Fc is a full-length 2-710 aa protein, carrying a human IgG1 Fc tag at the C-terminus, which is expressed by human 293 T cells (ACRO, China). Meanwhile, 3 kinds of probes containing GTM were used. GTM is a synthesized peptide according to the amino acid sequence of GTM. GTM-Fc is a Vγ4Vδ1- human IgG1 Fc fusion protein, of which the CDR3δ domain is replaced by the GTM sequence. γ4δ1(GTM)-TCR is a soluble γδTCR with GTM embedded in the TCR CDR3δ. All the results showed that the GTM didn’t bind to NCL directly. b. MST analysis of the binding activities between GTM-Fc and NCL-Fc proteins was conducted in four independent replicates, with a binding trend observed in only one instance (above). The binding assay between γ4δ1(GTM)-TCR and NCL proteins was conducted in three independent replicates, with a binding trend observed in only one instance (below). c. and d. The recombinant 2133-bp, full-length NCL gene was confirmed by agarose gel electrophoresis (c), and then, the construct was transfected into 293T cells for overexpression. EGFP expressed in fusion with NCL was observed under a microscope (d). e. The expression of the recombinant NCL-EGFP fusion protein was validated by Western blotting assays with anti-NCL and anti-EGFP antibodies. f. An immunoprecipitation assay was performed to confirm the specific interaction between NCL and the GTM-Fc probe. Scale bar 125μm.

Supplementary Data Fig 3. Expression of 12 stress-induced ectopic protein ligands of γδ T cells in tumor biopsy samples.


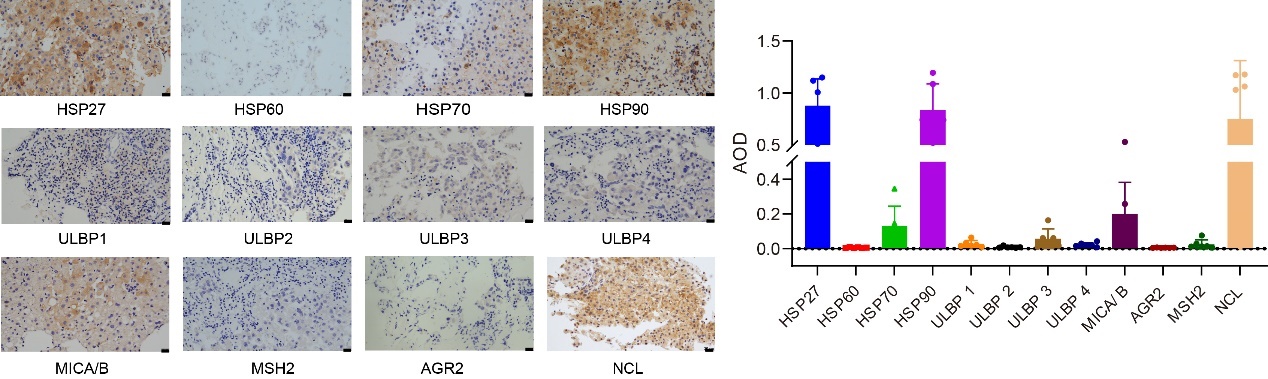


The expression of 12 protein ligands recognized by γδ T cells, namely, HSP27, HSP60, HSP70, HSP90, ULBP1, ULBP2, ULBP3, ULBP4, hMSH2, NCL, AGR2 and MICA/B, in tumor biopsy samples was detected by immunohistochemistry. A representative image for each protein ligand is shown on the left, and the average optical density (AOD) values in 6 different samples were analyzed and are shown on the right. Scale bar 20μm.

Supplementary Data Fig 4. Establishment of a menadione-induced cell stress model and its usage for stress-induced γδ TCR ligand detection.


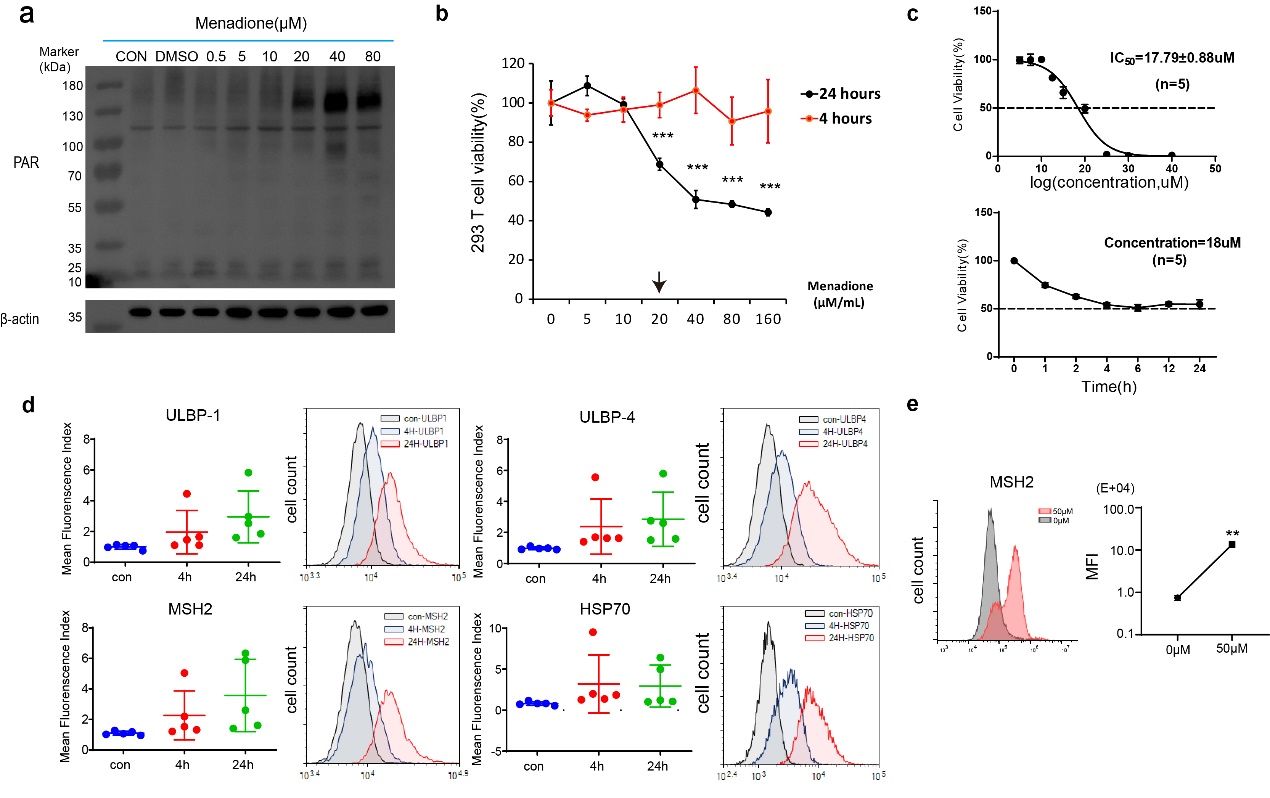


a. The characteristic enhanced modification of PAR after treatment with different concentrations of menadione was validated in NCI-H520 cell lysates by Western blotting. b and c. The effects of different treatment times and concentrations of menadione on the viability of 293T cells (b) and the tumor cell line NCI-H520 (c) were evaluated to establish a suitable cell stress model. d. The ectopic expression of several known stress-induced ligands was upregulated in menadione-treated 293T cells. e. Short-term high-dose menadione treatment also upregulated the ectopic expression of hMSH2 on tumor cells. Three independent experiments were conducted for statistical analysis, and representative results are shown. **, *p*< 0.01, ***, *p*< 0.001.

Supplementary Data Fig 5. Sorting of γδ T cells from PBMC.


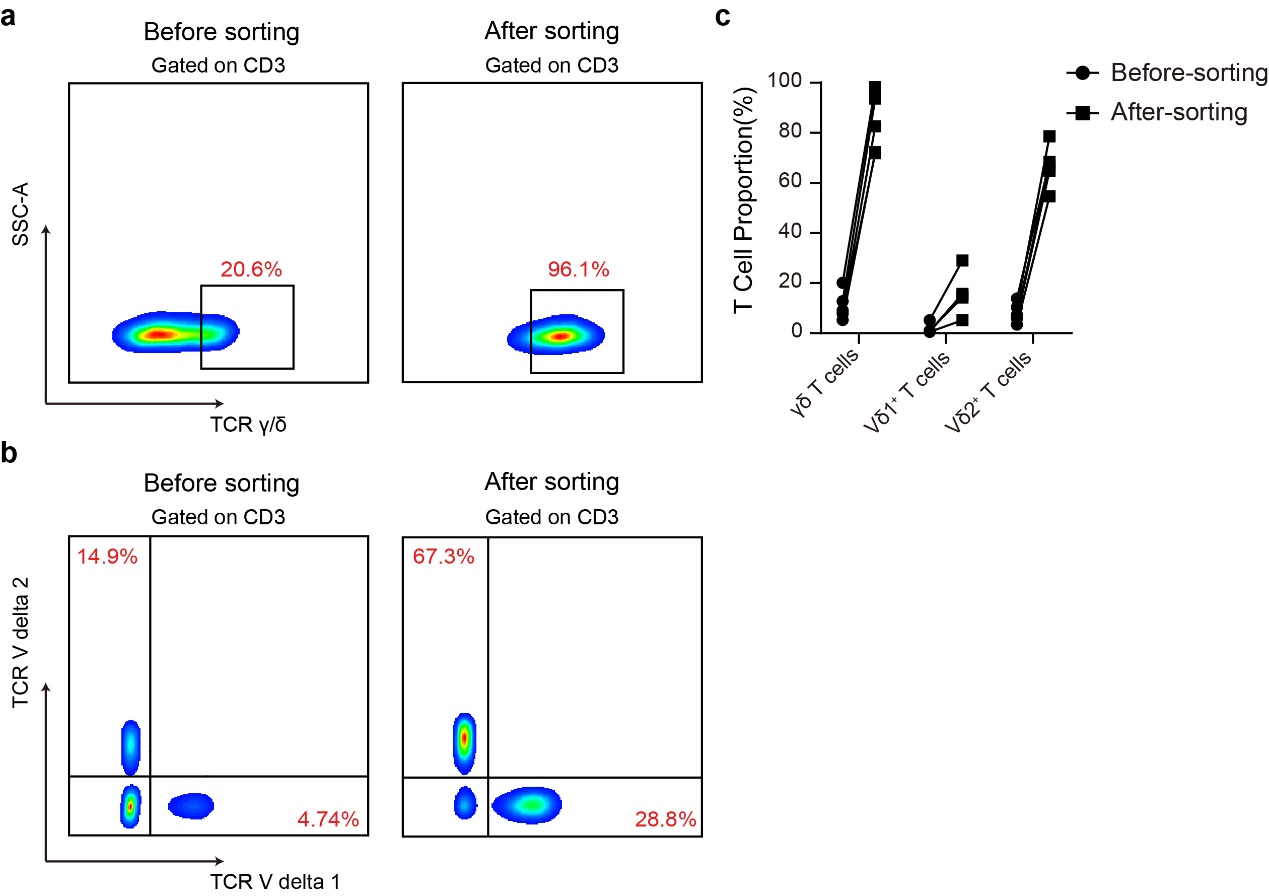


a. and b. Flow cytometry analysis of γδ T cells, Vδ1 and Vδ2 T cells before and after sorting of PBMCs. c. Statistical plot of T cells proportion before and after sorting.

Supplementary Data Fig 6. NCL promotes the expression of activation molecules CD25 and CD69 on Vδ1T cells.


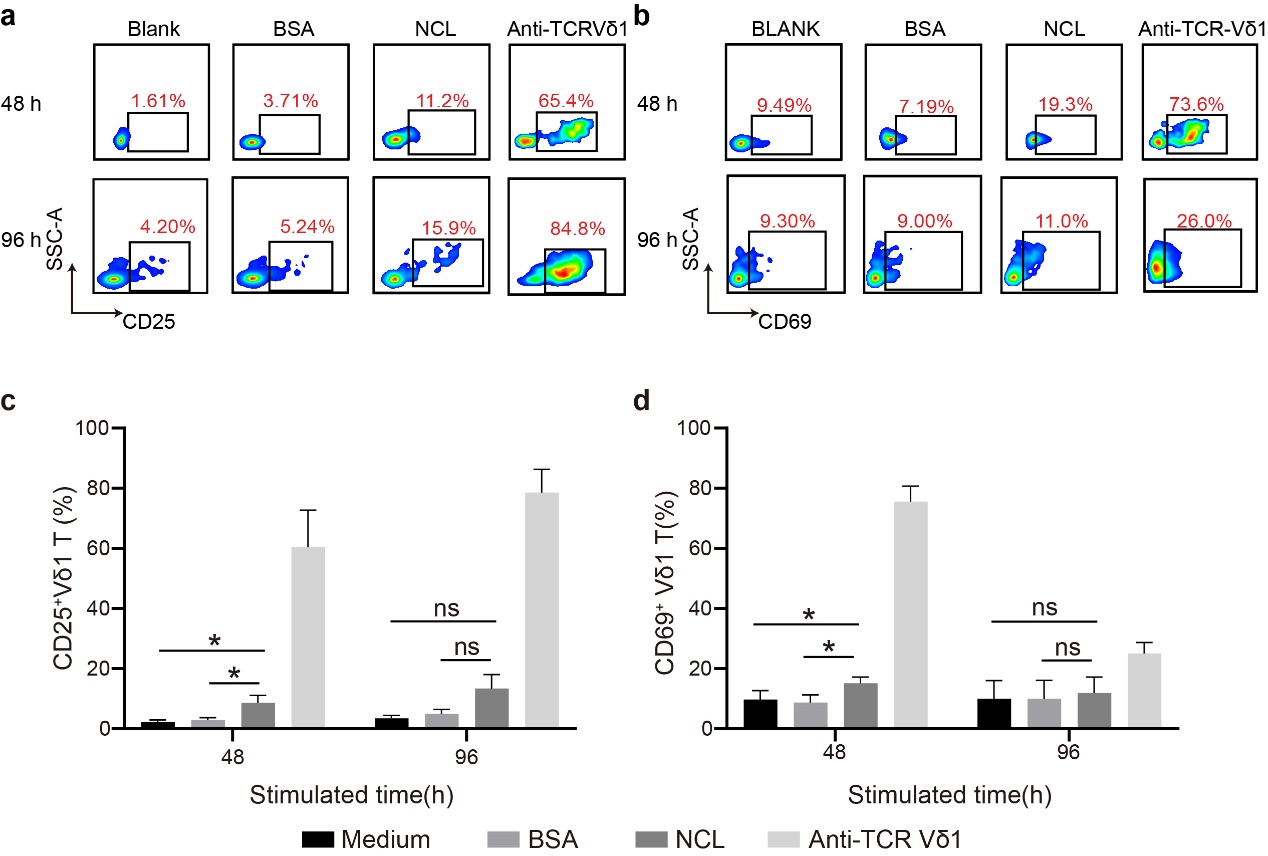


Flow cytometry analysis of CD25 (a/c) and CD69 (b/d) expression on Vδ1T cells after co-cultured with the recombinant NCL protein for 48 h and 96 h. 20μg/mL recombinant NCL was pre-coated for 2h and the sorted Vδ1T cell from PBMC was added then. BSA and anti-TCR Vδ1 antibody were used as negative control and positive control respectively. *, *p*< 0.05, ns: *p*> 0.05, no significance.

Supplementary Data Fig 7. Amplification Vδ1T cells from PBMC.


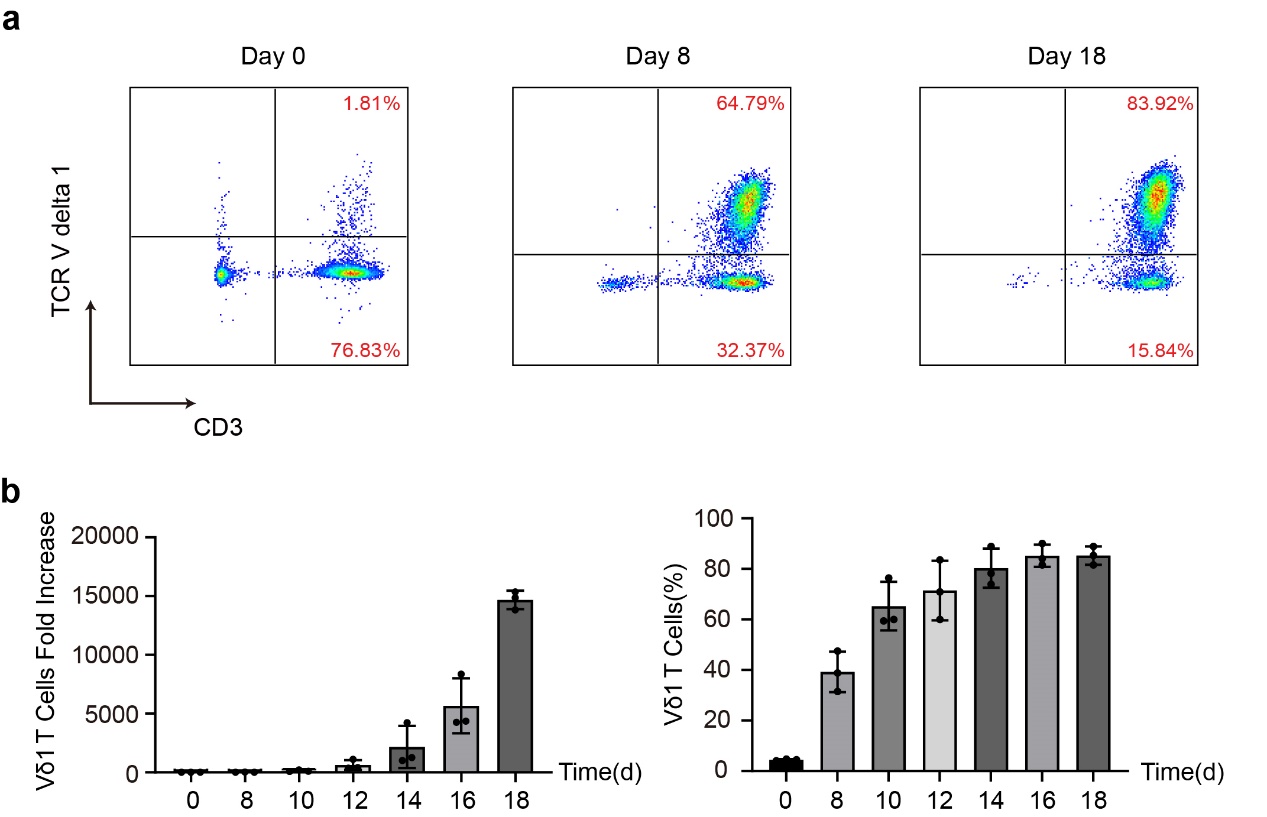


a. Representative flow chart of two-step method to selective expand and differentiate Vδ1 T cells from healthy donors, illustrating the growth of the proportion of Vδ1 T cells over time, b. Depicted are the fold increase (left) and fraction of Vδ1 T cells (right) at the indicated time points. Data are representative of three independent experiments.

Supplementary Data Fig 8. IFN-γ and IL-2 secretion of Vδ1T cells tested by ELISA


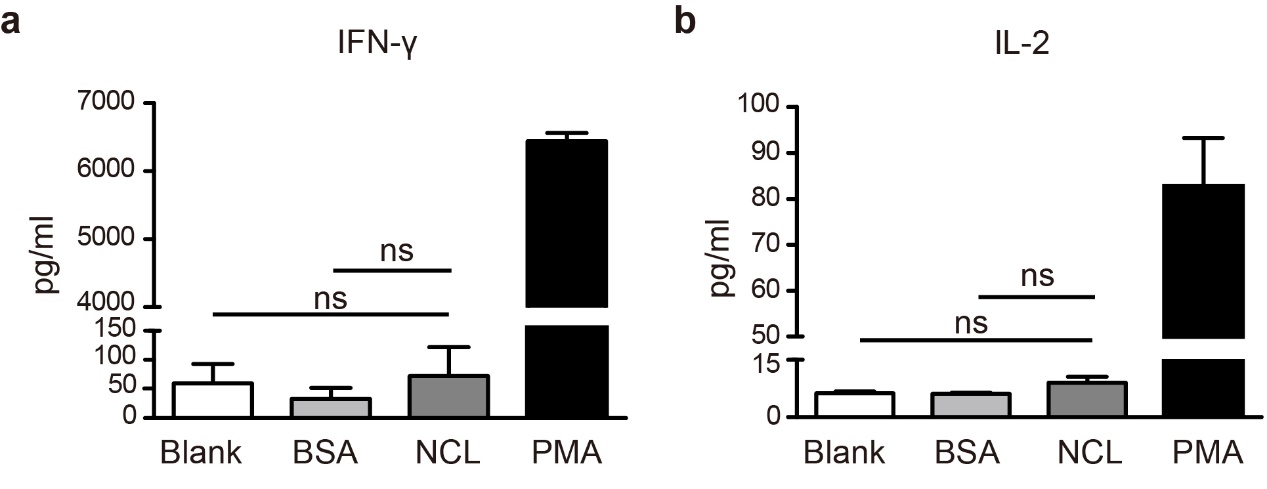


Resting Vδ1T cells were stimulated with NCL. The cells without added protein were used as a blank control, BSA was used as an irrelevant protein control, and stimulation with phorbol 12-myristate 13-acetate (PMA) + ionomycin (ION) was used as a positive control. Twenty-four hours after stimulating the Vδ1T cells, the levels of IFN-γ (a) and IL-2 (b) in the culture supernatant were detected by enzyme-linked immunosorbent assay (ELISA) (n = 4). ns: *p*> 0.05, no significance.

Supplementary Data Fig 9. FBXO2, with high expression in various human colorectal cancer cell lines, could specifically bind to the GTM-grafted soluble γδ TCR.


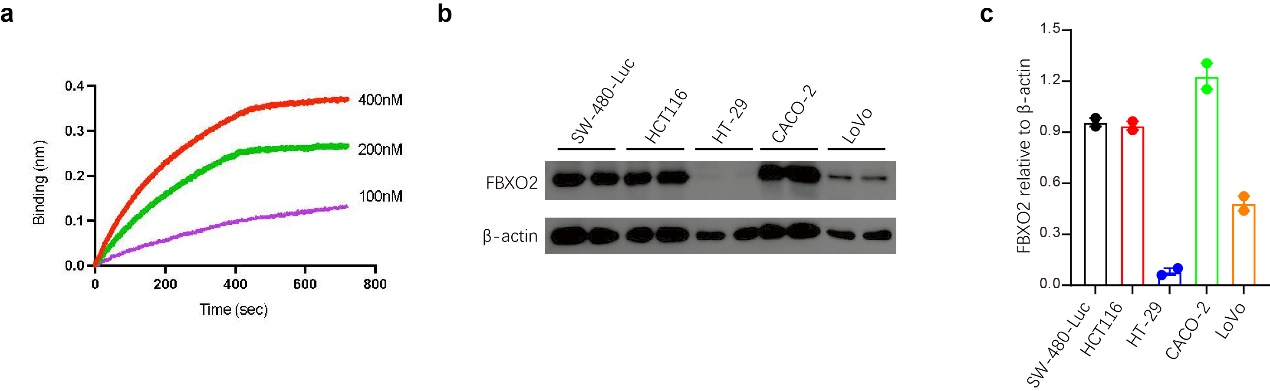


a. BLI assay to measure FBXO2 interacting with GTM-grafted soluble γδ TCR affinity. b. Western blotting was performed to evaluate the expression of FBXO2 in 5 colorectal carcinoma cell lines by using anti- FBXO2 antibodies. c. Quantification the expression of FBXO2 protein normalized to β- actin.

Supplementary Data Fig 10. Fluorescence detection for the expression of ZsGreen in lentiviral transduced T cells.


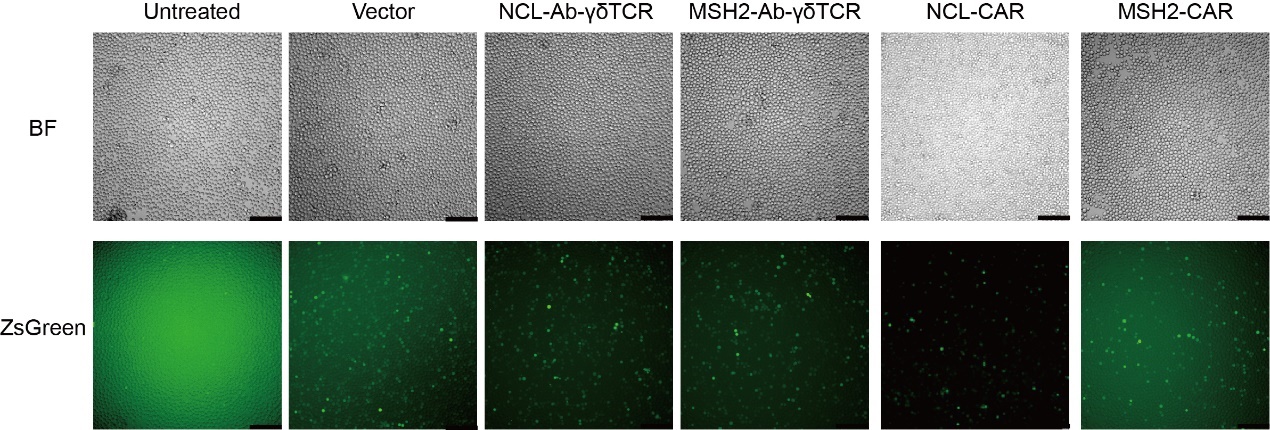


Fluorescence detection was performed to examine the expression of ZsGreen in T cells after 72 h of lentiviral transduction. Scale bar 100μm.

Supplementary Data Fig 11. Proliferation of Ab-γδ TCR/CAR-T cells in vitro.


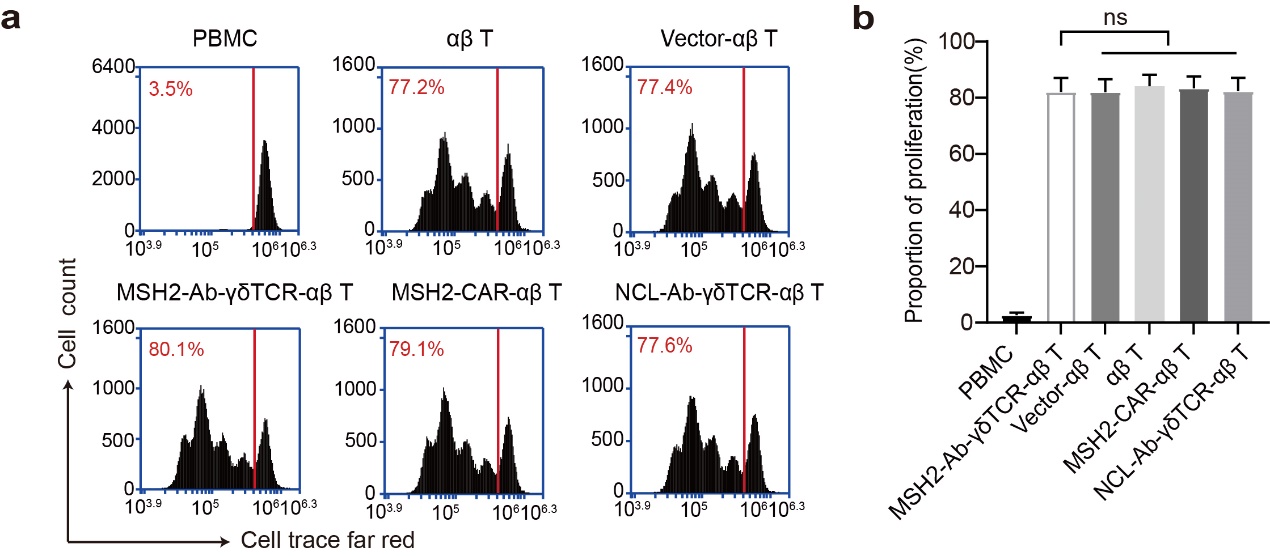


a. Flow cytometry was used to examine the proliferation of Ab-γδ TCR or CAR-T cells at 48 h after lentiviral transduction. b. The proportion of proliferated cells was determined. ns: *p*> 0.05, no significance.

Supplementary Data Fig 12. Tumor cytotoxicity of Ab-γδTCR/CAR-T cells in vitro.

**
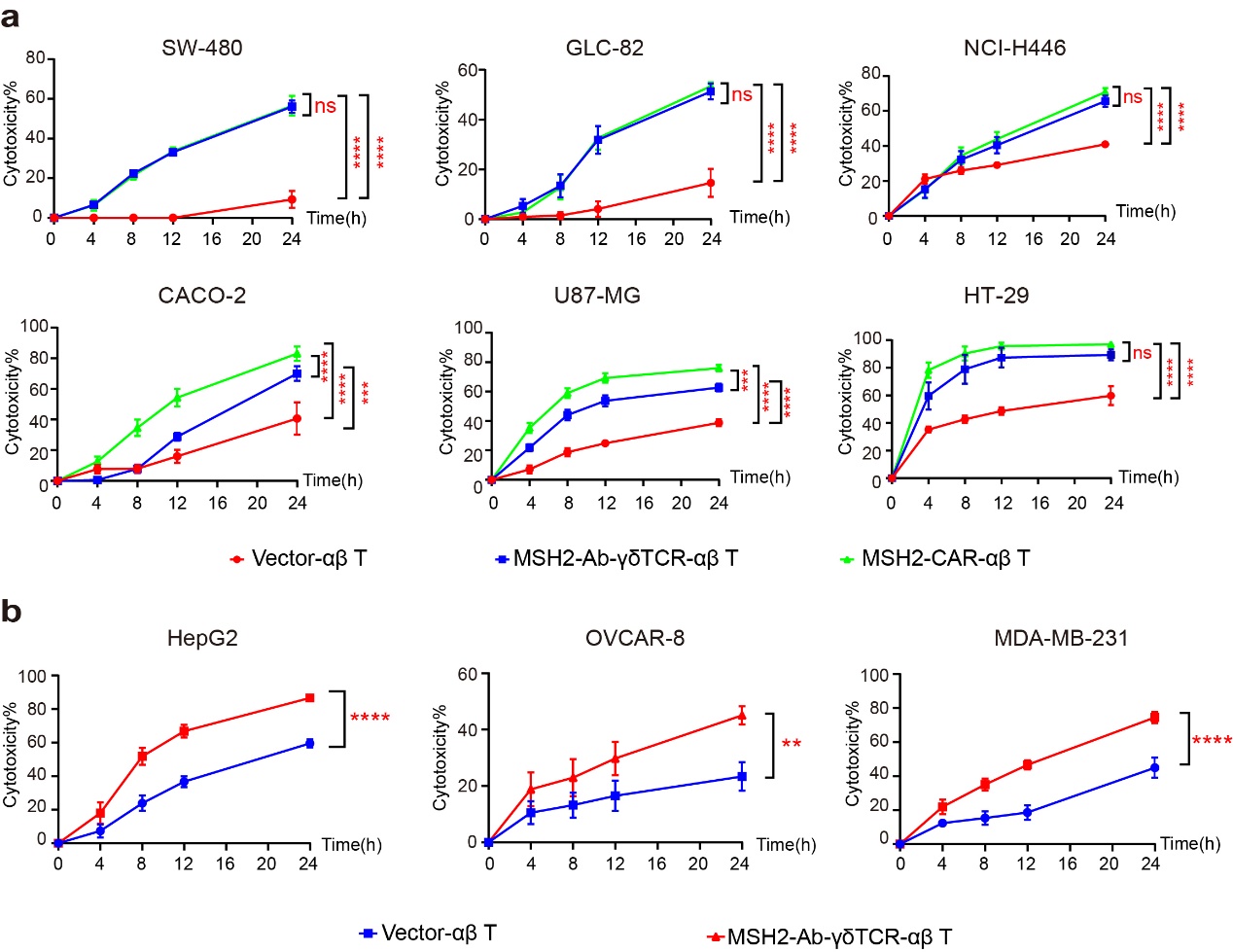
**

a and b. RTCA was performed to evaluate the cytotoxicity of hMSH2-Ab-γδ TCR/CAR-T cells (a) and NCL-Ab-γδ TCR/CAR-T cells (b) against different tumor cells. The plot shows the statistical data from effector cells and target cells co-cultured for 4, 8, 12, 16, 20 and 24 hours. The data are from 3 independent experiments and are expressed as the means ± SDs. ***, *p*< 0.001; ****, *p*<0.0001; ns: *p*> 0.05, no significance.

Supplementary Data Fig 13. Ectopic expression of hMSH2 and NCL in tumor cell lines.


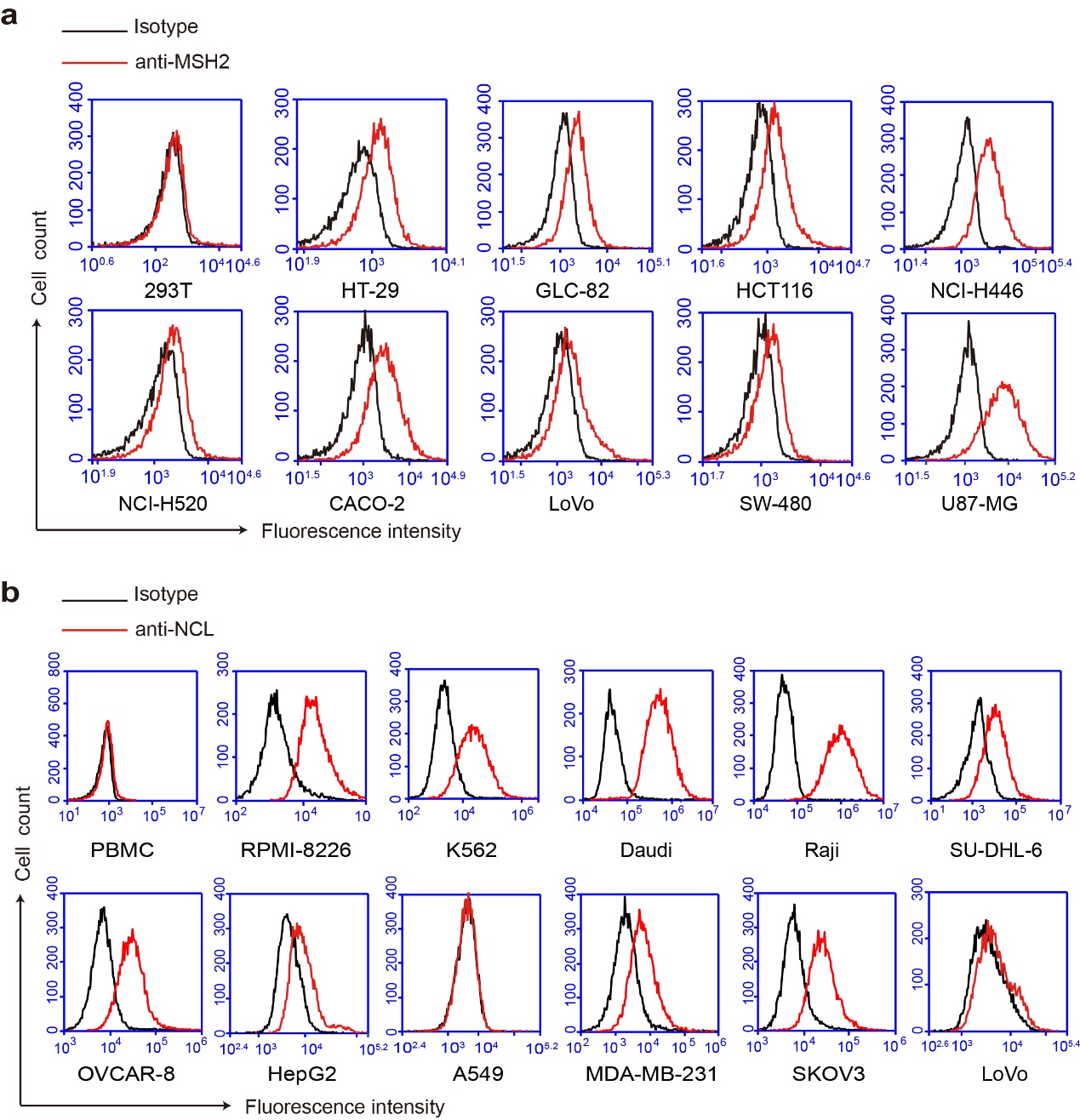


a and b. Flow cytometry was performed to analyze the expression levels of hMSH2 (a) and NCL (b) on the surface of different tumor cells. Representative plots from three independent experiments are shown.

Supplementary Data Fig 14. Verification of the specificity of Ab-γδTCR-αβT cells.


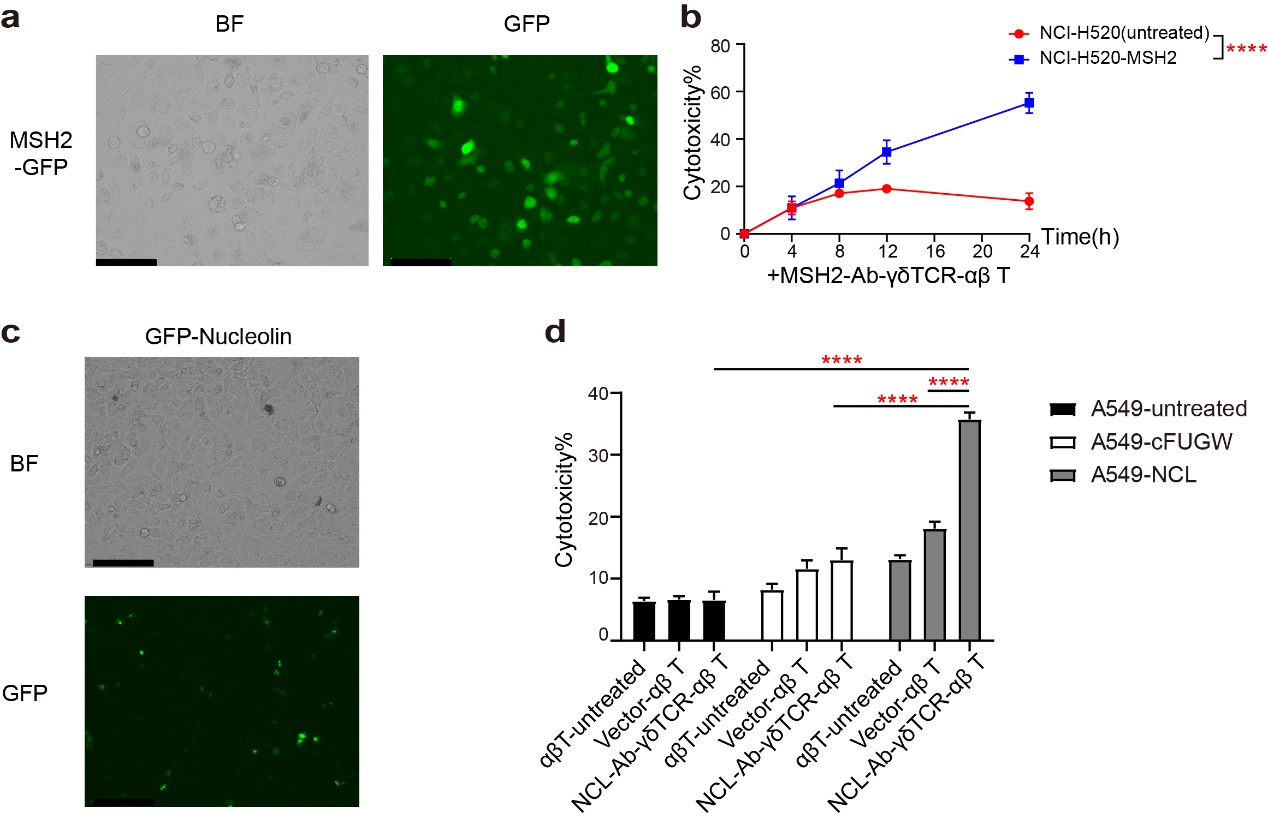


a. Fluorescence microscopy was performed to measure the GFP signal in NCI-H520 cells transfected with the plasmid hMSH2-GFP. Scale bar 125μm. b. Cytotoxicity of hMSH2-Ab-γδ TCR-αβ T cells against untreated NCI-H520 cells and hMSH2-overexpressing NCI-H520 cells (NCI-H520-hMSH2) cultured at an E:T ratio of 5:1 for 24 h. c. Fluorescence microscopy was performed to measure the GFP signal in A549 cells transfected with the plasmid GFP-Nucleolin. Scale bar 125μm. d. Cytotoxicity of effector cells (αβ T, MOCK-αβ T or NCL-Ab-γδ TCR-αβ T cells) against target cells (A549, A549-cFUGW or A549-NCL) cultured at an E:T ratio of 5:1 for 6 h. The data are from 3 independent experiments and are expressed as the means ± SDs. BF, bright field. ****, *p*<0.0001.

Supplementary Data Fig 15. The correlation analysis of stress-inducible ligands expression on different tumor cell lines and γδ T-cell cytotoxicity.


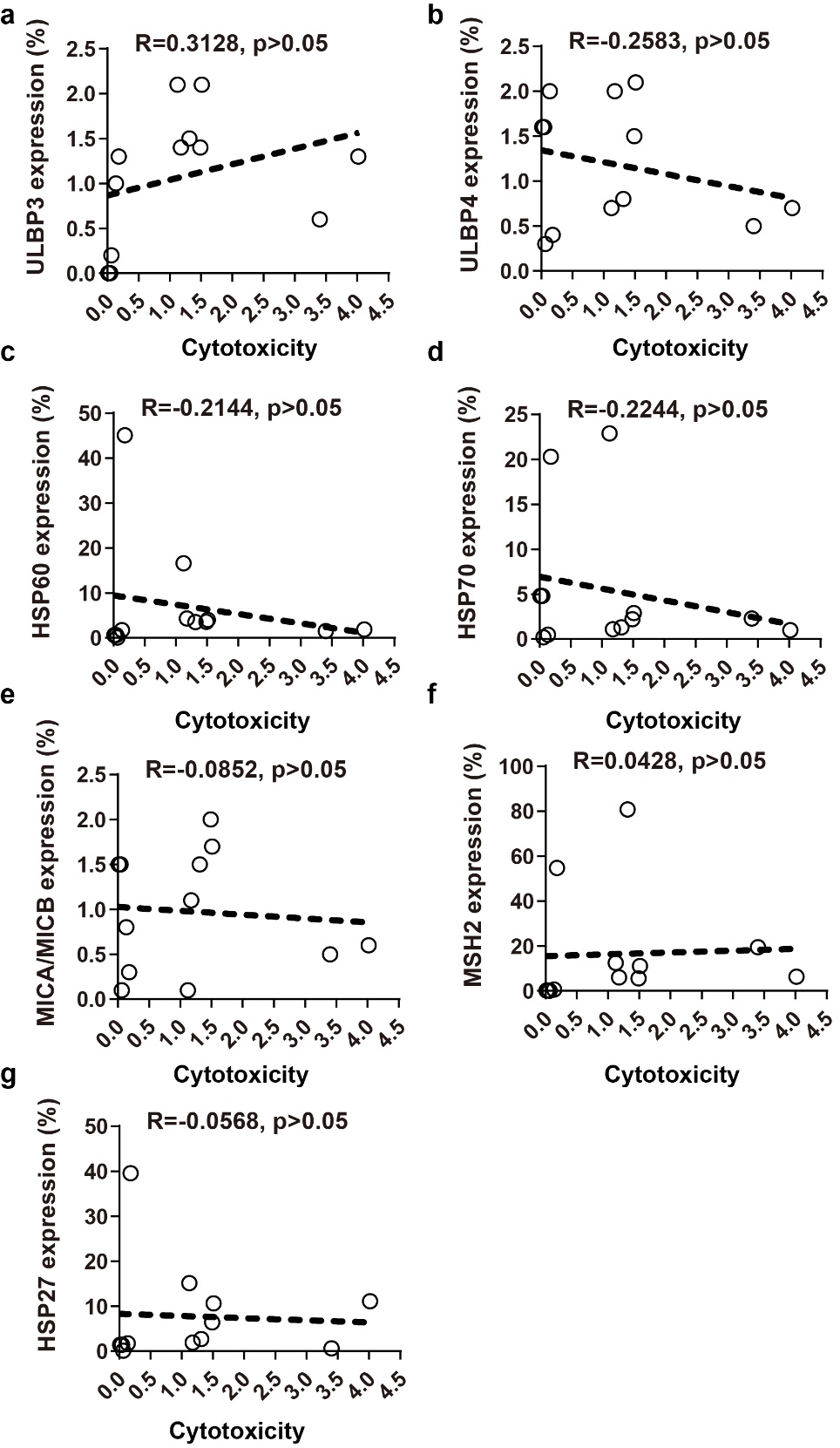


The expression of ULBP3 (a), ULBP4 (b), HSP90(c), HSP70(d), MICA/MICB(e), MSH2(f) and HSP27(g) were not correlated with γδ T-cell cytotoxicity.

Supplementary Data Fig 16. The difference in the expression of stress-induced ectopic proteins determines the sensitivity of tumor cells to γδ T-cell cytotoxicity.


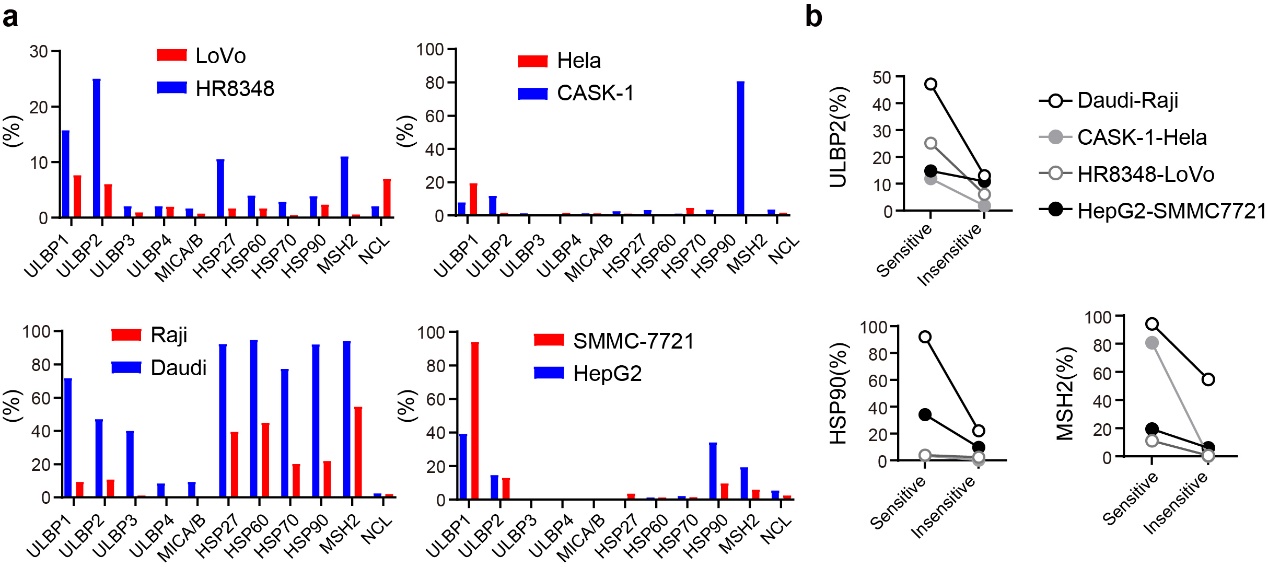


The expression levels of stress-inducible ligands were compared between 4 pairs of tumor cell lines, which were derived from the same tissue but significantly different in terms of their sensitivity to γδ T-cell cytotoxicity. a and b. Eleven stress-induced ectopic proteins were highly expressed in the γδ T-cell-sensitive tumor cell lines (a), and three of these ligands (ULBP2, HSP90 and hMSH2) exhibited consistent trends in each pair of γδ T-cell-sensitive and γδ T-cell-insensitive cell lines (b).

Supplementary Data Fig 17. The expression of the stress-inducible proteins ULBP1, ULBP2, HSP60 and MICA/B on irradiated tumor cells.


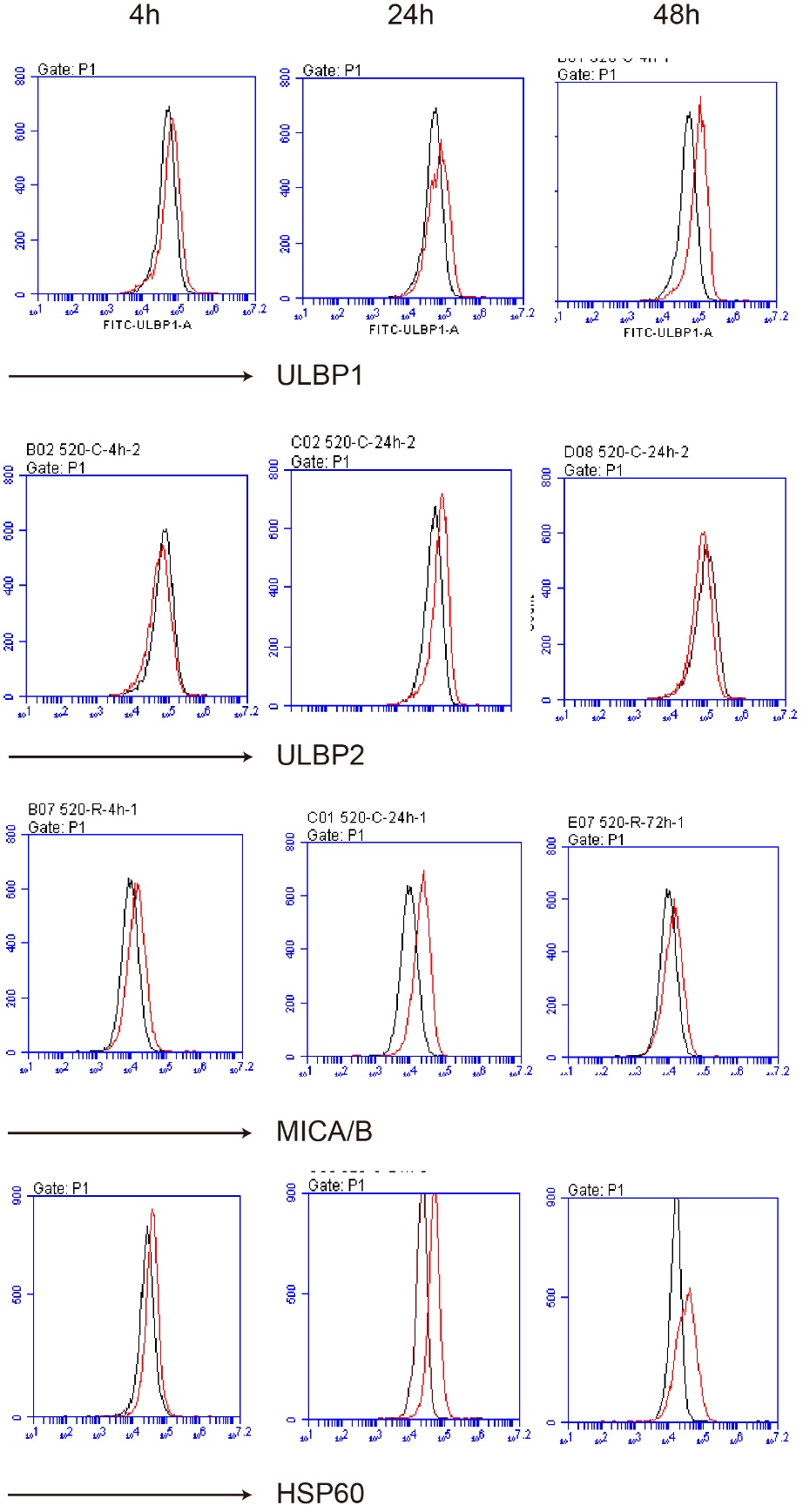


Representative flow cytometry results showing ULBP1, ULBP2, HSP60 and MICA/B expression on irradiated NCI-H 520 at 4h, 24h and 48h.

Supplementary Data Fig 18. The cytotoxicity of human γδ T cells against different irradiated tumor cell lines.


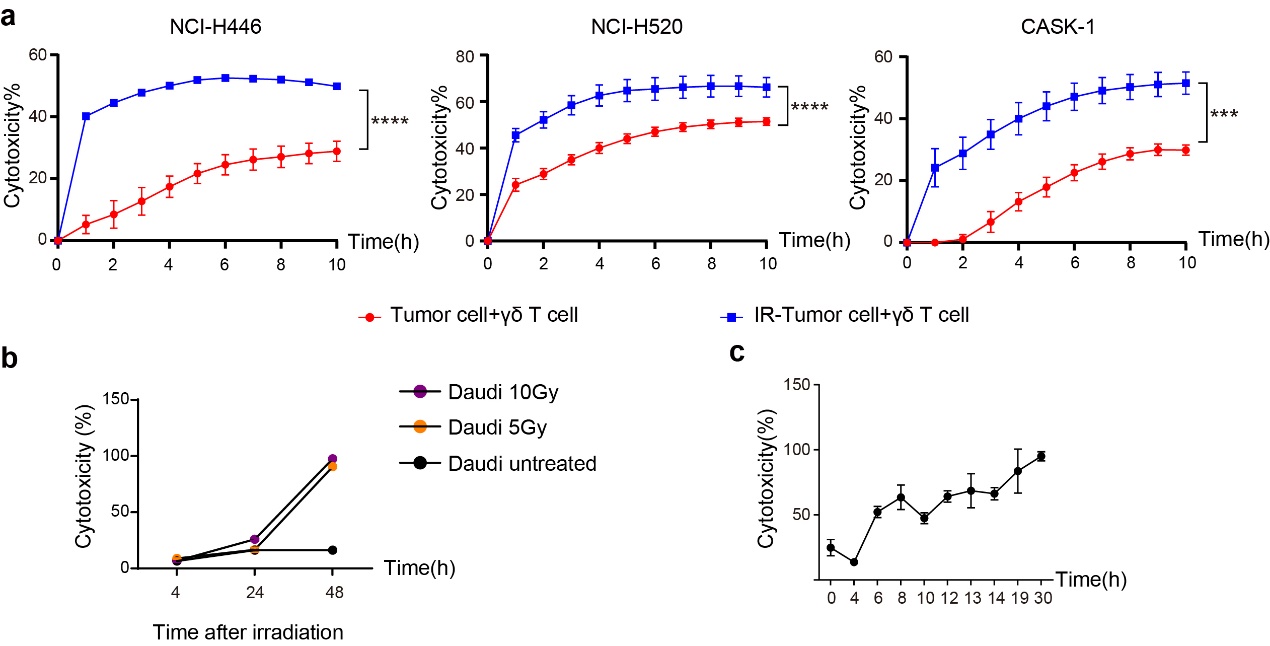


a. The cytotoxicity of human γδ T cells against irradiated tumor cells was significantly enhanced. The cytotoxicity of γδ T cells against three kinds of tumor cells treated with or without 5 Gy ionizing radiation was recorded by RTCA. b. The cytotoxicity of γδ T cells against Daudi treated with 5 or 10 Gy ionizing radiation was examined by LDH cytotoxicity assay. Untreated Daudi was used as control. c. The cytotoxicity of γδ T cells against RPMI-8226 treated with 5 Gy ionizing radia tion at different time points. The data are from 3 independent experiments and are expressed as the means ± SDs. ***, *p*< 0.001; ****, *p*<0.0001.
