# Supplementary material for: γδ T-cell autoresponses to ectopic membrane proteins: a new type of pattern recognition
Source: Cell Mol Immunol. 2025 Feb 13;22(4):356–70. doi: 10.1038/s41423-025-01258-x (PMC11955531; doi:10.1038/s41423-025-01258-x)
Supplement: Supplementary file 2 — Unprocessed original images of gels and western blots [file 41423_2025_1258_MOESM2_ESM.pdf]

Source Fig.4b

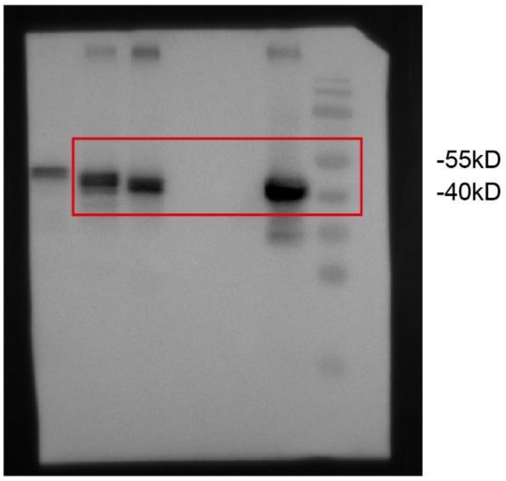

TCR δ

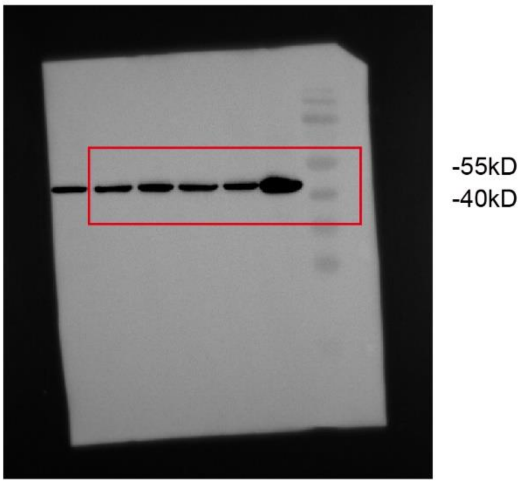

β-actin

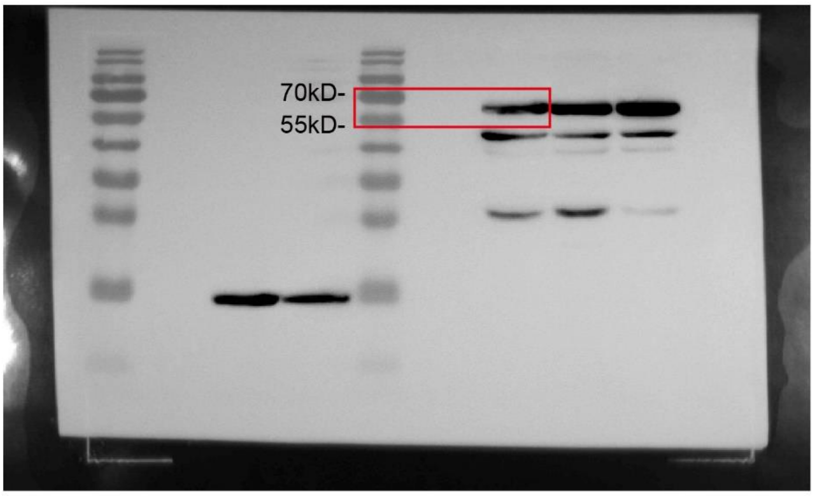

CD3 ζ

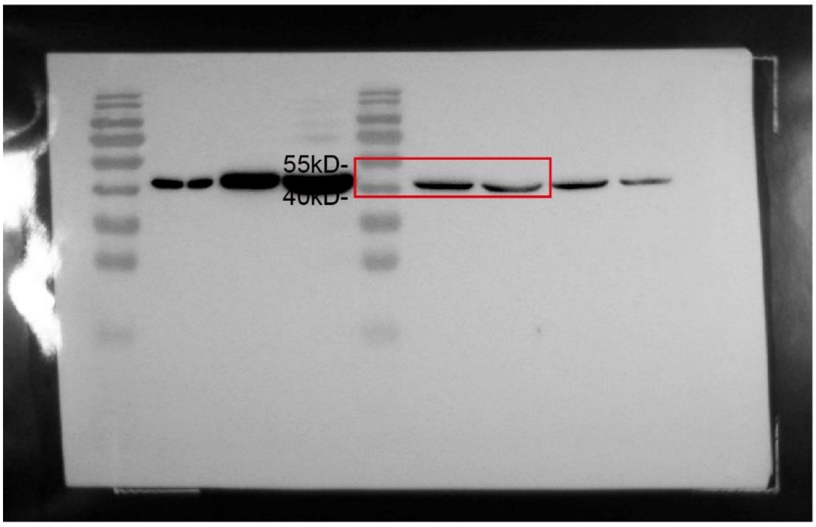

β-actin

Source Extended Data Fig.1

a

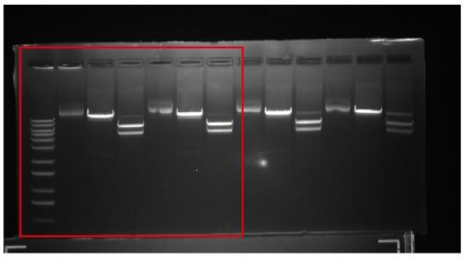

OT3

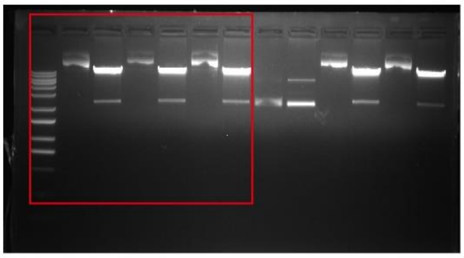

GTM

b

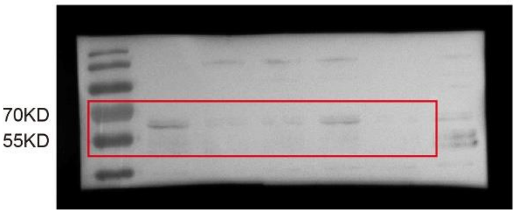

Flag-GFP

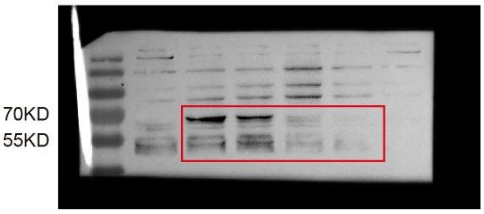

γδCAR-GFP

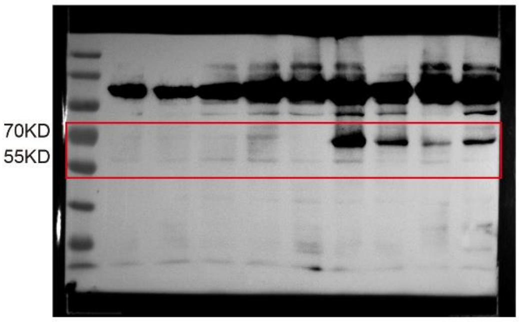

CD3 ζ

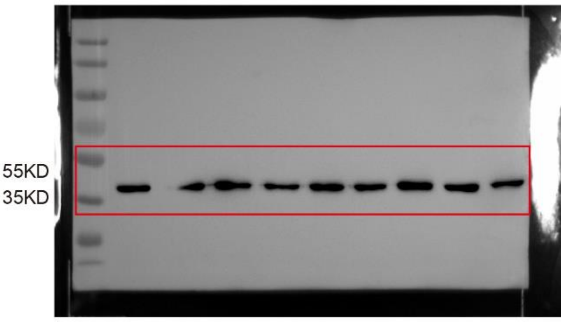

β-actin

Source Extended Data Fig.2

a

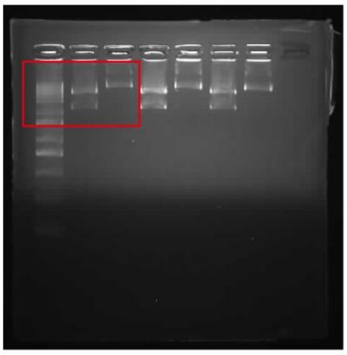

c

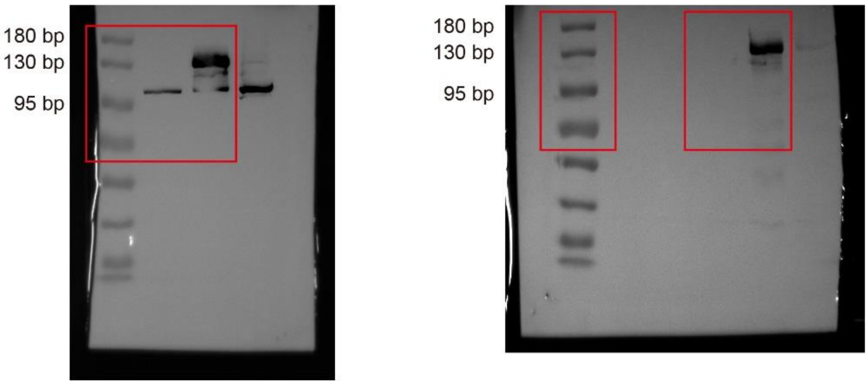

anti-NCL

anti-GFP

d

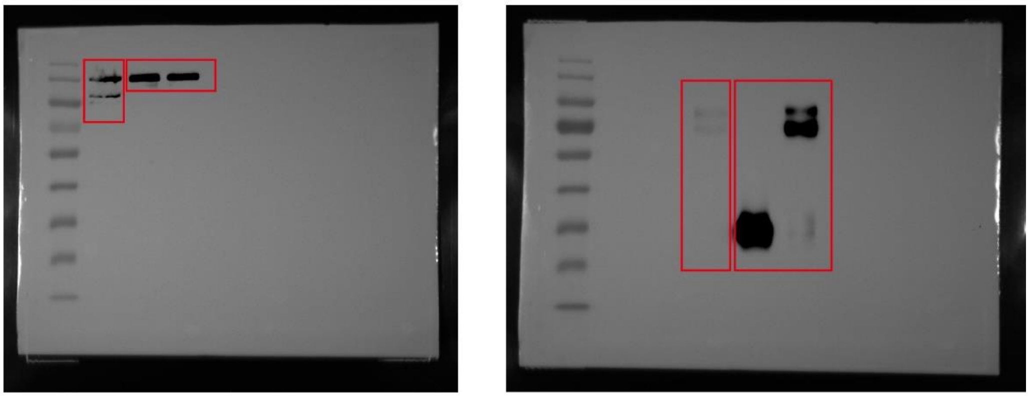

Source Extended Data Fig.4a

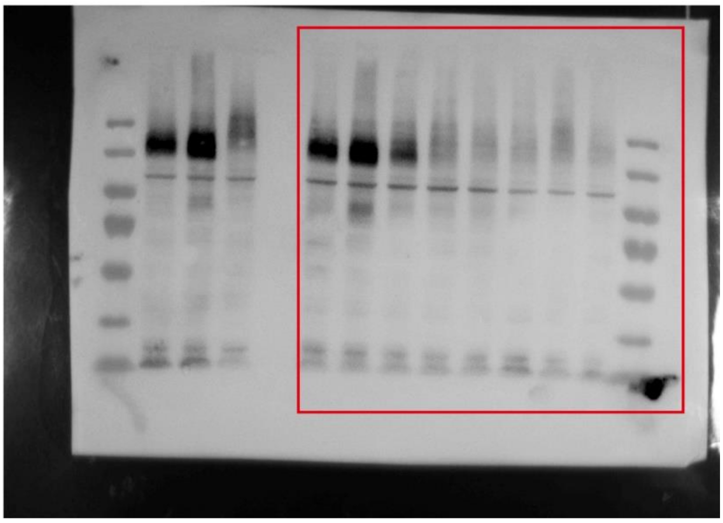

PAR

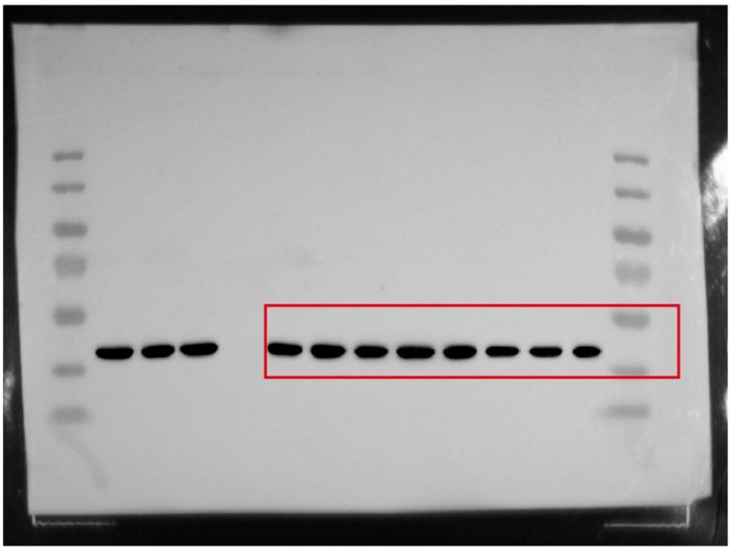

β-actin

Source Extended Data Fig.5b

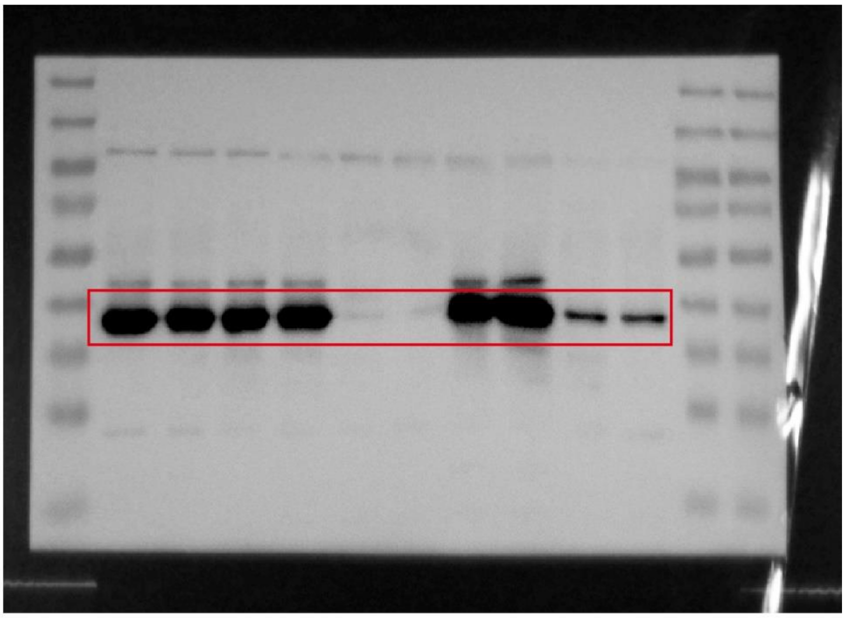

FBXO2

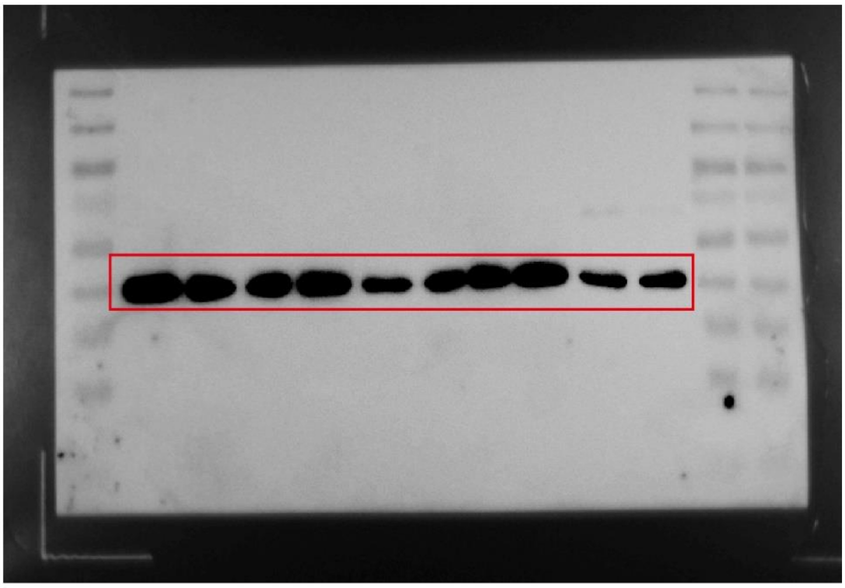

β-actin
